# Bacteriorhodopsin of purple membrane reverses anisotropy outside the pH range of proton pumping based on logic gate realization

**DOI:** 10.1038/s41598-024-80512-0

**Published:** 2024-11-27

**Authors:** Hamdy I. A. Mostafa, Abdo A. Elfiki

**Affiliations:** Department of Biophysics, Faculty of Science, Cairo University, Giza, 12613 Egypt

**Keywords:** Logic gate, Proton pump, Bacteriorhodopsin, Anisotropy, Dual-frequency biomaterial, Acid blue membrane, Alkaline purple membrane, Biophysics, Materials science, Nanoscience and technology

## Abstract

The bacteriorhodopsin of purple membrane is the first discovered light-sensing protein among ion transporting microbial rhodopsins, some of which (e.g. Archaerhodopsin 3) could be broadly used as tools in optogenetics having wide potential of medical applications. Since its discovery as early as in 1971, bacteriorhodopsin has attracted wide interests in nano-biotechnology, particularly in optoelectronics devices. Therefore, the present work has been motivated due to two topics; firstly, anisotropy demand became indispensible in bioelectronics; secondly, the stationary level of electric response in bacteriorhodopsin within the pH range of proton pumping (pH 3 – pH 10) implies, in turn, raising here a question about whether the electric anisotropy is implicated for reducing (or switching off) such level beyond such pH range. Noteworthy is that the purple membrane converts to blue form upon acidification, while to reddish purple form upon alkalization. In the present study, the acidic and alkaline forms of bacteriorhodopsin have exhibited most probable state of reversal for the dielectric anisotropy around pH 2.5 and pH 10.5, respectively. This is underscored by proposing a correlation seemingly found between disassembly of the crystalline structure of bacteriorhodopsin and the reversal of dielectric anisotropy, at such acidic and alkaline reversal pH’s, in terms of the essence of the crystalline lattice. Most importantly, the results have substantiated dual frequency characteristics and logic gate-based dielectric anisotropy reversal to bacteriorhodopsin, which may implicate it for potential applications in bioelectronics.

**Supplementary Information:**

The online version contains supplementary material available at 10.1038/s41598-024-80512-0.

## Introduction

The photochromic bacteriorhodospin (bR) is the sole protein embedded in the purple membrane (PM) of *Halobacterium salinarum* (*Hs*) to generate a proton electrochemical gradient across the membrane for cellular purposes like ATP synthesis^1^. It possesses unique photophysical properties lent it to be of considerable interest in technological applications^2–4^. Recently, there were many investigations to attempt, at least via enegineered bR (Highly Expressible bR)^5,6^, to open up its potential of optogenetics and medical applications in future^7–9^, to be like the proton pump archaerhodopsin 3 (Arch), which is nowadays best used in optogenetics^10^. The protein of bR consists of seven α-helices (A **–** G). The chromophore of bR monomer is a retinal moiety attached to a highly conserved lysine residue (Lys216) at the middle of the seventh helix (G) via a protonated Schiff-base linkage. The lipids connect three bR monomers to form trimers assembled in two-dimensional hexagonal crystalline structure in the form of a circular purple patches called purple membrane (PM)^11–13^. The contributing role of lipid-protein beside to the protein–protein interactions in connecting the bR trimer should be regarded not only in the structure of bR, but also in its function^11,12^. The bR functions as a light-driven outward proton pump through several intermediates written in a simple sequential form as: I_460_, J_625_, K_590_, L_550_, M_412_, N_550_ and O_640_^1,14^. It should be noted that the first two intermediates are the fastest ones: the sub-picosecond (~ 0.2 ps) excited I_460_ and the picosecond (~ 0.5 ps) hot vibrationally J_625_ intermediates^14^, followed by the K_590_ (~ 2 ps)^15^. Worthwhile to note that the color of bR changes to blue in K_590_, then to reddish purple in L_550_, to yellow in M_412_, to reddish purple in N_550_, to blue in O_640_ and then back to purple in bR_570_. This opsin cycle is concomitant to the initial photo-isomerization of retinal from all-*trans* to 13-*cis* configuration and then back to the all-*trans* conformer at the late of the bR photocycle^1^. Noteworthy is that other factors could also induce color changes in bR, for instances, the electric field (i.e. electrochromism or Stark effect^16^) and temperature (i.e. thermochromism^17^).The acidity and alkalinity induce color changes in bR, as well^18–20^. The pH-induced structural changes in bR might be correlated to the surface charge asymmetry of PM, as the cytoplasmic surface of PM is more negative than its extracellular surface above particular pH. In other words, this surface charge asymmetry^21^ is reversed at particular pH called pH_rev_^22,23^ (it is around pH 5 for wild-type bR^21–23^, which may be equivalent to its isoelectric point at pH 5.8^24–26^). Namely, below the pH_rev_, the extracellular surface became more negative instead. Therefore, electrically-oriented PM in suspension was found to turn itself to the opposite direction, and hence its name as “pH_rev_” was derived. Below pH_rev_, the permanent dipole moment of PM is lower in magnitude^21,23^. Even the bending of PM above and below the pH_rev_ is asymmetric: the PM bends towards its extracellular side above pH_rev_, while towards the cytoplasmic side below it^27,28^. However, there appear several aspects of the asymmetry in PM, for instances, the distribution of lipids in PM is also asymmetric^29^; the retinal position in PM is asymmetric^30^; the insertion of bR itself in PM is asymmetric^1^. Needless to say, these aspects of asymmetric PM do point to the heterogeneity in dielectric function throughout the thickness of the PM. Noteworthy is that the asymmetric distribution of charges brings about anisotropic electrostatic interactions, for an instance, the interactions of the major triglycosyl lipid (S-TGA-1) with protein were found to be asymmetric^29^, as well. These aspects of asymmetry might be pertinent to the anisotropy of bR in PM. Accordingly; pH-induced structural changes occurring in bR could be reflected as changes, for instance, in the dielectric function (direction-dependent) in bR. Therefore, before describing the present study, it would be advantageous in this connection to rationalize an intended concept for focusing on anisotropy to discern its importance to be studied here in the present work. The concept is categorized into the following two subsections.

### Anisotropy Alteration in purple membrane

It is well evidenced that there is charge anisotropy across the biological membranes^31^. It is most probably for purposes serving the membrane assembly^32^, the electric permittivity was found to be of larger values (comparable, at least, to the value of the solvent^33^) on the surface of the membrane (i.e. hydrophilic) than in the interior core of membrane (i.e. hydrophobic). For an instance, the aromatic amino acids are not in uniform distribution in bR so that they preferentially gather at the interface of membrane^29^. Such residues residing at the interface could interact with the head groups of lipids, making a hydrophobic mismatch^34^, induced by environmental factors like pH to be of profound consequences, mainly, at very low and very high pH values. However, changing the pH might results in alterations in the interfacial affinity of such aromatic residues (by virtue of its flanking) bringing presumably about different degrees of the hydrophobic mismatch. If so, the contribution from the mismatch of lipids’ tails (that are in contact with amino acid helices), may bring, in turns, about alterations in the structural ordering of bR assembly. Most important, such a preferential distribution of some residues, alongside with the non-uniform distributions, in general, of the other residues, might create optimized variations in the electric permittivity throughout the molecular structure of the membrane. Therefore, pH-induced changes in the optimized profile of this permittivity may be manifested as alterations in the structural ordering of PM, which could be considerable at acidic and alkaline pH’s, accounting for the dielectric anisotropy changes observed here. This rational may be supported by the finding that the non-uniform distribution of amino acid residues leads to anisotropic electrostatic interactions (i.e. directional electrostatics that is relevant to electric anisotropy), which play an essential role in the self-assembly of protein^35^. This might justify proposing here that pH-induced changes in such anisotropic interactions may make it possible for some new features (e.g. dipole–dipole interactions becoming long-range) to appear, which via their non-local effects throughout the whole PM, result ultimately in changes in the ordering of the crystalline structure of PM underlying consequences of the anisotropy changes. Actually, crystalline structure alterations were observed in diffraction studies^36^, in which the P622 crystal of bR was observed to change its size nearly at pH 3.5 and pH 10. Knowledge of the surface potential in PM at acidic and alkaline states could help signify the importance of the same two pH’s, as two groups were found to titrate at p*K*_a_ of 3.5 and p*K*_a_ of 10^37,38^. Based on the introduced concept above, a motivation for the present work is to record changes in the anisotropy of PM within and beyond such pH range (pH 3 – pH 10).

### Anisotropy Demand in Bioelectronics

The bioelectronics is an integration of a biological material with an electronic system, which has to be conducted via interfacing a biocompatible nanomaterial (e.g. carbon-based^39^) or molybdenum-based^40^ interfaces); all the three components organize the biohybrid electronic system opening up widely potential applications, particularly from sensing perspectives^39,41–43^. For instance, in the technological research field of bR, concepts inspired from the cell membrane capacity have actually enabled designing bR-based biocapacitor, which represents an integration of the bR biomaterial with a nanomaterial acting as a nanochannel. The principle of such biocapacitor relies on converting the electric signal, generated upon illuminating bR with light, into well-regulated square signal^44^. The latter could, besides, be exploited on the other hand as a logic input (i.e. oscillates regularly between two binary digits: 1 for maximum and 0 for minimum voltage) to logic electronic circuits^44^. Speaking of which, a bR-like proton pump, proteorhodopsin (pR), was utilized as a light-powered biocapacitor^45^, as well. First of all, it should be noted that designing electronic systems entails awareness with the anisotropy implying, in turn, to understand anisotropy of biomaterials being integrated with electronic components on the one hand. Specification of the bio-nano interactions (between bio- and nano-materials) might entail the anisotropy state of both bio- and nano-materials to be more desired on the other hand. In general, the electric anisotropy, as such, concerns direction-dependent conductivity, dielectric permittivity, and permeability. Worthwhile to note that bR possesses liquid–crystal-like ferroelectric behavior^46^ and pyroelectric behavior^47^.These electric behaviors, together with possessing large permanent electric dipole moment normal to PM^22^, could allow bR to possess dielectric anisotropy, as well. The anisotropic properties reflect anisotropic structural features, as structure–property relationships is controllable by anisotropy^48^. Moreover, the preparation of anisotropic hydrogels (nanomaterial composites), as an instance, strongly substantiates that the anisotropic electric properties, amongst other anisotropic properties (e.g. mechanical), are of key aspects designing desired bioelectronics to conduct programmable output^49^. Despite being challenge, the electric anisotropy could be programmable, merely by altering the nanomaterial composite relying on the fact that the extent of alteration has profound consequences on the degree of such programmed anisotropy^49–52^. Even the interactions in the gel composite would be anisotropic^53^ if long-term bioelectronics would be desired, as the anisotropic gel does not suffer aging ensuing from the thermodynamic instability of the isotropic conventional gel^52^ as a nanomaterial. As to the biomaterial like bR, the dispersion of bR-carbon nanotube hybrids, for instance, showed stability in the pH range of (pH 4.5 – pH 9)^54^ in fluorescence studies. Outside a comparable pH range of (pH 2.5 – pH 10.5), the results here have showed that bR reverses the anisotropy, implying a clue for the bio-nano interactions being presumably controlled by anisotropy. Accordingly, the anisotropy seems to become so essential for both bio- and nano-materials in bioelectronics that the concept of programmable anisotropy could lead to advances in bioelectronics^50,52,55^. In this regard, realization of the electric anisotropy to be programed in bR is not elusive in future anisotropy-based bioelectronics.

In the present work, logic gate realization of the electric anisotropy to be reversed in wild-type bR has been introduced, which may be of potential applications in what is called programmable bioelectronics^41,56,57^. This study has concerned with two commonly used arguments in bioelectronics, particularly in sense technology, viz*.* pH^57^ and crossover frequency (*f*_C_)^58,59^. In a wide range of pH below and above the pH_rev_ (from pH 1.59 to pH 11.2), the dielectric anisotropy studies in the frequency range (42 – 3 × 10^5^ Hz), alongside with the emission (with UV excitation at 280 nm) in the range (300 **–** 500 nm), have been conducted. The latter has to be conducted, as perturbations could possibly occur in the retinal environment because of the pH effect that does imply changes in the spectral features of the fluorescence of bR to occur significantly^54^. The results here have showed that the dielectric anisotropy reverses its sign, based on logic gate realization, at the acidic and alkaline forms of bR, around pH 2.5 and pH 10.5, respectively. Outside this range, the proton pumping is well-known to be reduced considerably^18,24,38,60–62^. The pH at which the anisotropy reverses can be defined as crossover pH (pH_C_). No such reversal in dielectric anisotropy was observed here at the so-called pH_rev_. Similarly, the frequency, at which the dielectric anisotropy reversal occurs, is defined as crossover frequency (*f*_C_) and the material that exhibits such reversal is referred to as “dual frequency” material^58,59^. On the basis of these definitions, the study here represents experimental results of pH dependences (i.e. input of logic gate) and frequency dependences (i.e. input of logic gate) of the dielectric anisotropy (i.e. output of logic gate) in bR. In summary, bR reverses the sign of dielectric anisotropy solely at pH just beyond (i.e. logic digit “1”) the two pH limits (i.e. called here as pH_C_) of the pH range of proton pumping provided the operating frequency (*f*) should be above the crossover frequency (*f*_C_) (i.e. logic digit “1”) realizing bR as an “AND” logic gate. An “OR” logic gate could be realized, as well. In general, these two arguments (one chemical, i.e. pH (or pH_C_), and the other electrical, i.e. *f* or (*f*_C_)) have been employed as input stimuli (which may be programmable) to the bR biomaterial (acting as “AND” (or as “OR”) logic gate, respectively) to get an electrical output (represented by one magnitude only for the dielectric anisotropy, i.e. > 0, 0, < 0). To conclude, the paper presents dual frequency and logic gate characteristics to bR, which may be of technical relevance.

## Methods

### Purple membrane orientation

The bR of PM of *Halobacterium salinarum* S9 is purchased from Sigma-Aldrich Co. The PM is basically prepared according to a well-established procedure described by the discoverers of bR, Oesterhelt and Stoeckenius^63^. Diluted suspension of 5 μM of PM was prepared according to molar absorption coefficient of 63,000 cm^-1^.M^-1^, at wavelength of 568 nm for light-adapted state of bR.

The electric orientation of PM fragments was carried out by applying 25 V/cm DC electric potential field across the orienting electrodes^22,64^. The orientation should be carried above pH_rev_, as the surface charge asymmetry of PM below and above pH_rev_ is of opposite sign, and the permanent dipole moment of PM becomes lower below pH_rev_. Accordingly, the PM fragment is aligned with its surfaces parallel to the face of the electrode. In the meanwhile, the PM fragments were immobilized, in polyacrylamide matrix, simultaneously during its orientation by electric field^65^. Rotating the PM sample by 90° (vertically) could enhance the measurements to be carried out in parallel or perpendicular to the direction of the permanent dipole moment of bR (i.e. out-of-plane or in-plane, respectively).

### Measurements

The passive electric properties of bR were recorded by applying 0.3 V AC signal to a cell containing the PM sample. The cell is made of platinized platinum electrodes of cell constant (K = 0.03 m^-1^). By applying the mode of constant voltage provided by Hioki 3532 LCR analyzer, the dielectric absorption spectra could be recorded in the frequency (*f*) range (42 – 3 × 10^6^ Hz). The analyzer is interfaced to a personal computer and the measurements are carried out through the Hioki 9261-test fixture connected to the LCR analyzer. The resistance (R) and capacitance (C) of the PM sample, as an equivalent parallel RC element, are simultaneously recorded in both orthogonal directions. The dielectric permittivity (ε′) and the dissipation factor (or loss tangent factor, tan δ) are determined from the measured values of (R) and (C) as follows^66^: (ε′) = C**/**(Kε_o_) and tan δ = 1**/**(ωRC), respectively, where ω = 2π*f*, the angular frequency in radian; K, the cell constant in m^-1^ and ε_o_, the permittivity of free space (8.85 × 10^–12^ F**/**m). The data were accounted for the electrode polarization and ionic conductance. Furthermore, the measuring cell was made so appropriate that favors electrode polarization minimization^66^.

Shimadzu spectrofluorometer (RF-150X) was used to record the steady-excitation fluorescence spectra in the range of (300 – 500 nm). The fluorescence from the randomly-oriented PM sample in suspension was collected at a right angle to the excitation beam at 280 nm.

The dielectric absorption measurements were carried out in dependence of pH in a wide range (pH 1.59 – pH 11.20) for immobilized PM, whereas the emission measurements were carried out in the range (pH 2.40 – pH 11.20) for PM in suspension, to avoid PM aggregation that usually occurs below pH 2.40. A Jenco digital pH-meter (Model-5001) was used to adjust the pH of interest using the system of HCl**/**NaOH and phosphate buffer. Mono-, di- , and tri-basic sodium phosphate buffers of 1 mM in concentration are used to adjust pH throughout the wide range of pH from pH 1.59 to pH 11.20. All measurements were conducted at 27 °C.

### Data evaluation

The curve fitting of pH dependences was carried out according to Eq. 1 of Henderson- Hasselbalch:

1 $${y}_{1}\left(pH\right)={A}_{i}+\frac{\left({A}_{max}-{A}_{i}\right)}{\left[1+{10}^{n\left(pH-{pK}_{a}\right)}\right]}$$

where (*A*_*i*_) and (*A*_*max*_) are assigned to the initial and maximum (or final) limits, respectively, of the pH transition; the difference between these limits gives the magnitude of the transition (Δ*A*) in the measured response (*y*_*1*_). The value of (*n*) is assigned to the number of protons that are transferred in the pH transition the center of which lies at p*K*_a_ of the transition; the value of (*n*) is related to the slope of the transition at p*K*_a_.

Multiplicative combination of Eq. 1 could be done according to the following^67^:

2 $${y}_{2}\left(pH\right)=\left(\frac{\left({A}_{max}-{A}_{i}\right)}{\left[1+{10}^{-{n}_{1}\left(pH-{pK}_{a1}\right)}\right]}+{A}_{i}\right)\left(\frac{\left(1-{A}_{f}\right)}{\left[1+{10}^{{n}_{2}\left(pH-{pK}_{a2}\right)}\right]}+{A}_{f}\right)$$

Or alternatively, according to the following equation, particularly if both the initial and final limits (*A*_*i*_ and *A*_*f*_) of the bell-shape curve, which comprises such two multiplicative (i.e. opposite) titrations, have to be put to zero ensuring one maximum (*A*_*max*_) only for the bell-shape curve^67^.

3 $${y}_{3}\left(pH\right)=\left(\frac{1}{\left[1+{10}^{-{n}_{1}\left(pH-{pK}_{a1}\right)}\right]}\right)\left(\frac{1}{\left[1+{10}^{{n}_{2}\left(pH-{pK}_{a2}\right)}\right]}\right)$$

The same meaning is given to (*n*_*1*_ and *pK*_*a1*_) for the one side and (*n*_*2*_ and *pK*_*a2*_) for the other side of the bell-shaped curve, as well as to the responses (*y*_*2*_ and *y*_*3*_). Likewise, the same meaning is given for two titrations, which are not opposite to each other, but rather additive (i.e. sequential). This sequential composite curve could be fitted by additive combination of Eq. 1, for two (or more) sequential titrations according to the following equation:

4 $${y}_{4}\left(pH\right)={A}_{0}+\left(\frac{\Delta {A}_{1}}{\left[1+{10}^{{n}_{1}\left(pH-{pK}_{a1}\right)}\right]}\right)+\left(\frac{\Delta {A}_{2}}{\left[1+{10}^{{n}_{2}\left(pH-{pK}_{a2}\right)}\right]}\right)$$

The first and second parentheses are for the first and second additive titrations, respectively, plus constant background (i.e. the offset *A*_*0*_); the other parameters have the same meaning as given above.

Curve fitting of difference biphasic spectra belonging to the Δ(tan δ) could be done according to the Lorentz distribution of two components (i.e. double peaks), represented by (*y*_*5*_), as follows^68^:

5 $${y}_{5}\left(x\right)={A}_{0}+{A}_{1}\frac{{\upgamma }_{1}^{2}}{{\left(x-{xc}_{1}\right)}^{2}+{\upgamma }_{1}^{2}}+{A}_{2}\frac{{\upgamma }_{2}^{2}}{{\left(x-{xc}_{2}\right)}^{2}+{\upgamma }_{2}^{2}}$$

where *A*_*0*_; the offset value, *A*_*1*_ and *A*_*2*_; the Amplitude, *xc*_*1*_ and *xc*_*2*_; the center, and γ_*1*_ and γ_*2*_; the half-width at half-maximum (HWHM) of the first and second Lorentzian peaks, respectively. It is worth noting here that *x* = log (*f*); *f* is in Hz.

Curve fitting of fluorescence spectra could be done rather according to the Lorentzian distribution of a single band superimposed on a little quadratic background (of coefficients: A_0_ , A_01_, A_02_), represented by (*y*_*6*_), as follows^68^:

6 $${y}_{6}\left(x\right)={A}_{0}+{A}_{01}{x}+{A}_{02}{x}^{2}+A\frac{{\upgamma }^{2}}{{\left(x-xc\right)}^{2}+{\upgamma }^{2}}$$

The value of (γ) should be multiplied by 2 to get (*W*); the full width at half-maximum (FWHM). The parameters *A* and *xc* refer to the amplitude and the position of the band. It is worth noting here that *x* = wavelength (λ) in nm.

Polynomial curve fitting of the anisotropy factor could be done according to the following equation, with a polynomial of degree n = 8^68^, represented by (*y*_*7*_):

7 $${y}_{7}\left(x\right)={a}_{0}+{a}_{1}{x}++{a}_{2}{x}^{2}+{a}_{3}{x}^{3}+\dots +{a}_{n}{x}^{n}$$

Among the non-linear curves, the above equations are among the simplest ones, which could be fitted here by the implementation of the Levenberg–Marquardt algorithm^69^ to perform non-linear least squares fitting to estimate non-linear parameters in an iterative programming. How to use this algorithm is found elsewhere^70^. However, several computing software packages enable customizing (e.g. multiplicative or additive (or both) combinations in one equation) the non-linear curve fitting of these equations as user-defined equations.

## Results

### Most probable reversal in anisotropy

**Fig. 1:**
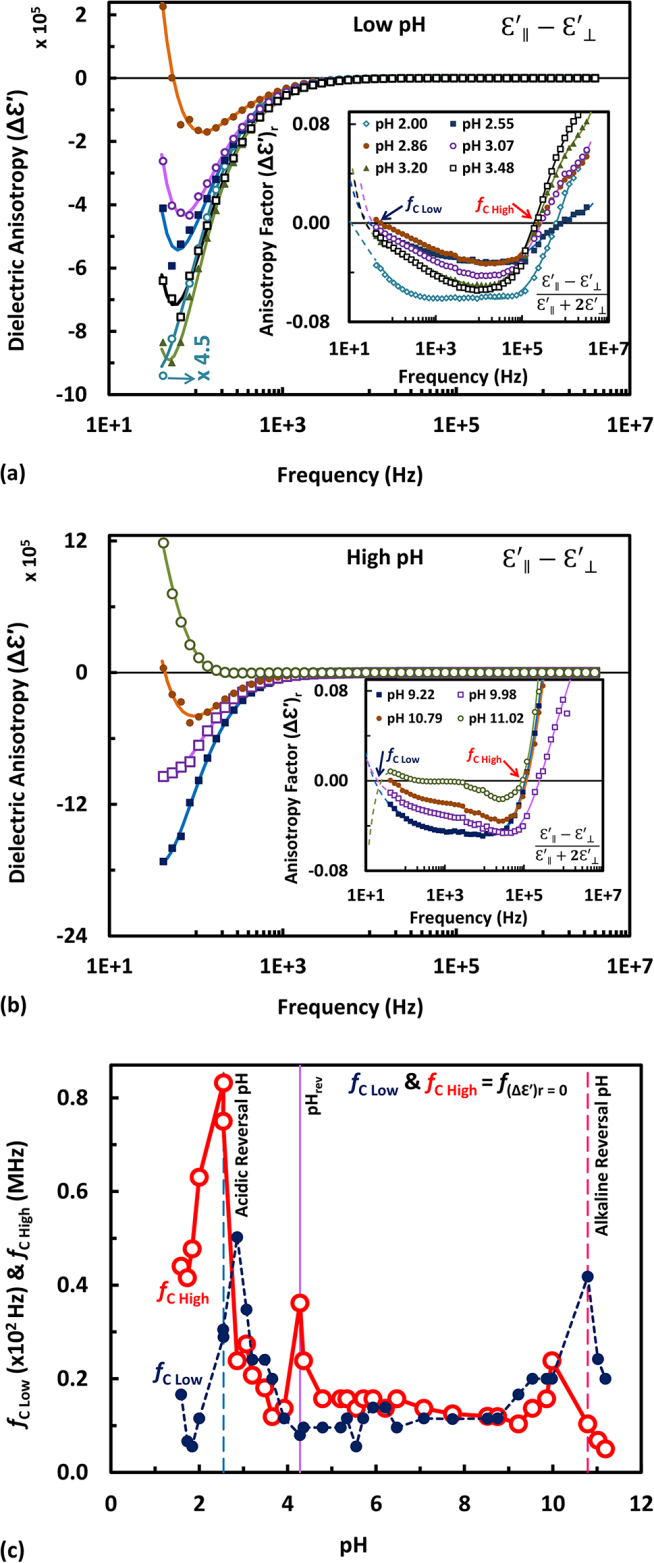
The pH dependence of the crossover frequency (*f*_C Low_) and (*f*_C High_) at low and high frequency, respectively. The frequency dependence of the dielectric anisotropy (Δε′) of immobilized PM at low pH in (**a**) and at high pH in (**b**). The inset represents the frequency dependence of anisotropy factor (Δε′)_r_, in accordance to the inset formula. The crossover frequencies (*f*_C Low_) and (*f*_C High_) are derived from the inset in (**a**) and (**b**), respectively, by polynomial extrapolation of the data back to cross the abscissa, as indicated by the dashed back extensions. Note that the data at pH 2.00 in (**a**) should be multiplied by a factor of 4.5 for scaling purposes. Note the anomalous behavior of the trace at pH 11.02 in (**b**). In addition, four pH sets are depicted in Fig. S1, Fig. S2, Fig. S3 and Fig. S4 in the “Supplementary Information”. The pH dependence at low frequency (or at high frequency) is shown in (**c**) exploring two significant pH’s; marked by vertical dashed lines at around pH 2.85 (or pH 2.6) and around pH 10.7 (or pH 10.1), at which the reversal of dielectric anisotropy displays a maximum, in addition to pH_rev_ marked by vertical solid line around pH 5.5 (or pH 4.3), at which immobilized oriented PM attempts to turn itself to the opposite direction.

The dielectric anisotropy (Δε′) is defined as the difference between the dielectric permittivities measured in parallel and perpendicular directions^58,59^ as Δε′ = (ε′_||_—ε′_⊥_). This dielectric anisotropy, demonstrated in Fig. 1 (as well as in Fig. S1 up to Fig. S4 in the “Supplementary Information”), should be normalized firstly. The normalized dielectric anisotropy, or simply anisotropy factor (Δε′)_r_ is written as [(ε′_||_—ε′_⊥_) **/** (ε′_||_+ 2ε′_⊥_)]. The frequency dependence of dielectric anisotropy factor (Δε′)_r_ at low and high pH’s is shown in the inset of both Fig. 1a and Fig. 1b, respectively, in addition to the insets of (Fig. S1 **–** Fig. S4) in the “Supplementary Information”. The anisotropy factor representation versus frequency here is to find the so-called crossover frequency (*f*_C_) at which the dielectric anisotropy equals zero upon its crossing, for instance, from positive to negative sign at low frequencies or from negative to positive at high frequencies, when the operating frequency (*f*) increases. Accordingly, the sign designation of the parallel and perpendicular directions has to be assigned properly. Generally, the dielectric anisotropy factor, as shown in the inset of both Fig. 1a and Fig. 1b (in addition to that of Fig. S1 up to Fig. S4), is initially negative at intermediate values of frequency, whereas positive at both low and high frequencies. While this reversal in sign can be clear at low frequencies (but at some values of pH), it is not clear whatsoever, at high frequencies, owing to the scale resolution. For this reason, the dielectric anisotropy had to be normalized firstly. Notice that the frequency at the crossover point is labeled as (*f*_C Low_) and (*f*_C High_) referring to the crossover frequency occurring at low and high frequencies, respectively. For the low frequency range, it is noted that the sign reversal is seen only at some pH values; while at the other pH’s it would appear at frequencies but beyond the lower limit of the present range of frequency. It could be possible, through the curve fitting procedure, to back extend the data to the frequency axis and obtain such crossover frequency. This could be performed by the polynomial curve fitting of data according to Eq. 7 with degree n = 8 extrapolated in small frequency range (seen by dashed lines in the inset of Fig. 1a and Fig. 1b, in addition to that of Fig. S1 – Fig. S4) so that the crossing point with the abscissa could be obtained merely by navigating through the data points, with satisfied precision. However, the more the population numbers of fitted data, the more the precision in getting the intercept with the abscissa (at zero ordinate). There would be two intercepts only: (*f*_C Low_) and (*f*_C High_) within the frequency range under investigation, for all the pH values of interest, except for extreme alkaline pH’s (e.g. pH 11.02 in the inset of Fig. 1b and pH 11.19 in the inset of Fig. S4) which show anomaly in the back extension, the reason of which is not clear, but it may be justified as follows. It is owing probably to the crystal being found to be of less pronounced assembly at pH ≥ 10^71^, in addition to being drastic changes in the polarization anisotropy reported to occur^72^, in the pH range of (pH 9 – pH 11), which could induce the crystal to become deteriorated (above pH 12)^73^. The pH dependence of the dual frequency characteristic for both (*f*_C Low_) and (*f*_C High_) has been depicted in Fig. 1c. This dependence has displayed two peaks (one at the acidic and the other at the alkaline side), in addition to a third one lying between these two peaks. The third that lies around pH 5, as shown in Fig. 1c, is reminiscent to what is so-called pH_rev_, at which electrically-oriented PM (in suspension) turns itself, due to reversal of its surface charge asymmetry, to become facing the opposite orienting electrode instead. Interestingly, the pH_rev_ is enclosed by the two reversal pH’s, at which the acidic and alkaline reversals of dielectric anisotropy of PM occur. Such reversal of dielectric anisotropy has been found to be of high probability to occur at acidic pH around 2.5, at alkaline pH around 10.5, and at the pH_rev_ around 5, as inferred from Fig. 1c (and later from Fig. 3c), i.e. it is least probable to occur at almost all pH’s, whereas most probable to occur solely at these particular (or discrete) pH’s. The values of most probable crossover frequency at these three peaks (acidic, pH_rev_ and alkaline) have been found to be 50, 5 and 40 Hz for (*f*_C Low_) respectively, while 0.85, 0.38 and 0.25 MHz for (*f*_C High_), respectively, as shown in Fig. 1c. Such discrete pH’s may seem to be significant, as it is sufficed to note that the color of the membrane differs quite at such three discrete pH’s, namely it is blue at the acidic reversal pH (around 2.5), neutral purple at nearly neutral pH_rev_ (around 5) and almost reddish purple at the alkaline reversal pH (around 10.5). This does not necessarily mean that the color change (i.e. opsin shift) can be considered as a determinant to the reversal occurring in the anisotropy. Rather, the driving factors (e.g. structural changes in bR), causing an opsin shift to appear, might be the determinant of such reversal. The structural changes of the protein resulting in retinal conformational changes are one of the factors that contribute to the opsin shift to appear^74^. The changes in anisotropy correlate with the pH-induced structural changes in bR, but not directly with the color change. However, this does not exclude the color change from becoming an indicator for the reversal of anisotropy.

**Fig. 2:**
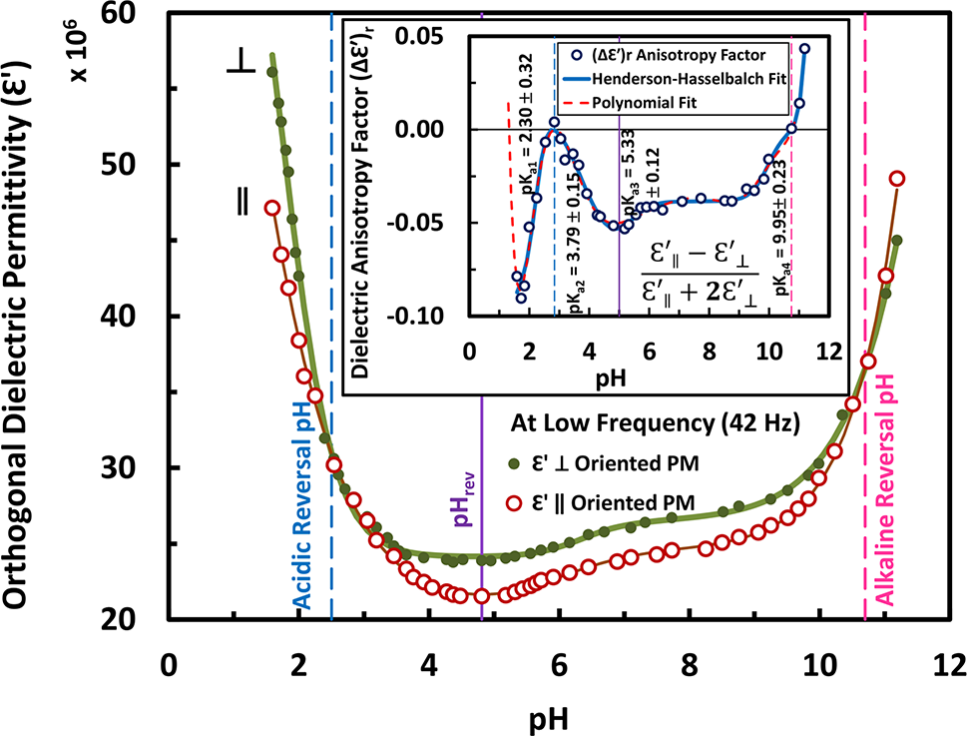
The pH dependence of orthogonal dielectric permittivity (ε′) of immobilized PM at low frequency (e.g. 42 Hz), in addition to the dielectric anisotropy factor (Δε′)_r_ in the inset, explore a significance for the same two pH’s (as mentioned previously in Fig. 1c) at around pH 2.8 and around pH 10.7, marked by vertical dashed lines (i.e. encloses the pH range of proton pumping), in addition to the pH_rev_ marked by vertical solid line around pH 5. In the inset, the solid line going through the data points are due to the Henderson-Hasselbalch fitting to four p*K*_a_’s, while the dashed line is due to polynomial fitting. The p*K*_a_’s and errors are shown in the inset.

For such important assignment of the three reversal pH’s, the pH dependence of dielectric permittivity in orthogonal directions (ε′_||_ and ε′_⊥_), for instance, at low frequencies (e.g. 42 Hz and 50 Hz) had to be intended in this regard, as shown in Fig. 2 and Fig. S5 in the “Supplementary Information”, respectively. These frequencies were intended not only because the dispersion of the permanent electric dipole moment (i.e. determinant of the dielectric anisotropy) occurs in the frequency range of (10 Hz -100 Hz)^22,64,75^, but also because both (42 Hz and 50 Hz) represent the maximum crossover frequencies at the alkaline and acidic reversal pH’s, respectively (refer to Fig. 1c). Such pH dependence at single frequency displays, as shown in Fig. 2 at 42 Hz (in addition to Fig. S5 at 50 Hz in the “Supplementary Information”), the two traces (ε′_||_ & ε′_⊥_) in a state of coincidence (i.e. tangential to each other associated with little overlapping) at pH 2.5. The above results extend and confirm previous studies made at high frequency (i.e. 1 MHz)^76^. The pH dependence of the anisotropy factor have showed, in Fig. 2 (inset) together with Fig. S5 (inset) in the “Supplementary Information”, four p*K*_a_’s titrations, according to the Henderson-Hasselbalch fitting, the detailed assignment of which is found elsewhere^9,18,20,38^. These four titrations could be fitted according to the summing of Eq. 2 (for the first two multiplicative titrations) and Eq. 1 (for each of the last two additive titrations). This combination of Henderson-Haselbalch fitting could be almost the same with a polynomial of degree nine. Indeed, the data in Fig. 2 (inset) at 42 Hz, in addition to Fig. S5 (inset) at 50 Hz in the “Supplementary Information”, were well fitted also to the polynomial function (dashed fitted line), which can suggest that the crossing to positive dielectric anisotropy would occur but at much lower pH beyond the lower limit of the pH range of interest, as indicated from the trend of the back extension of the fitted dashed line. The latter has been intended to realize an unequivocal reversal at low pH’s (i.e. pH_C_). It is worth mentioning that the acidic pH dependence of the anisotropy factor (see the inset of Fig. 2 herein, in addition to the inset of Fig. S5 in the “Supplementary Information”) exhibits a trend, which likens to that of the transition temperature (but in the reciprocal form) concerning the main melting of PM in thermal studies^77^ (Fig. 1 therein), indicating a thermal stability for the acid purple form of bR in contrast to the instability of its acid blue form at around pH 2.5. The same spoken can be given to the transition temperature in the alkaline pH dependence as indicated with DSC studies^78^ (Fig. 2 therein). Worthwhile to note, in this regard, that the acid purple became a chloride pump, whereas the acid blue is still a proton pump^19^.

### Consistence of (H_Fract_) with (f_C_) at discrete pH’s (i.e. pH_C_)

**Fig. 3:**
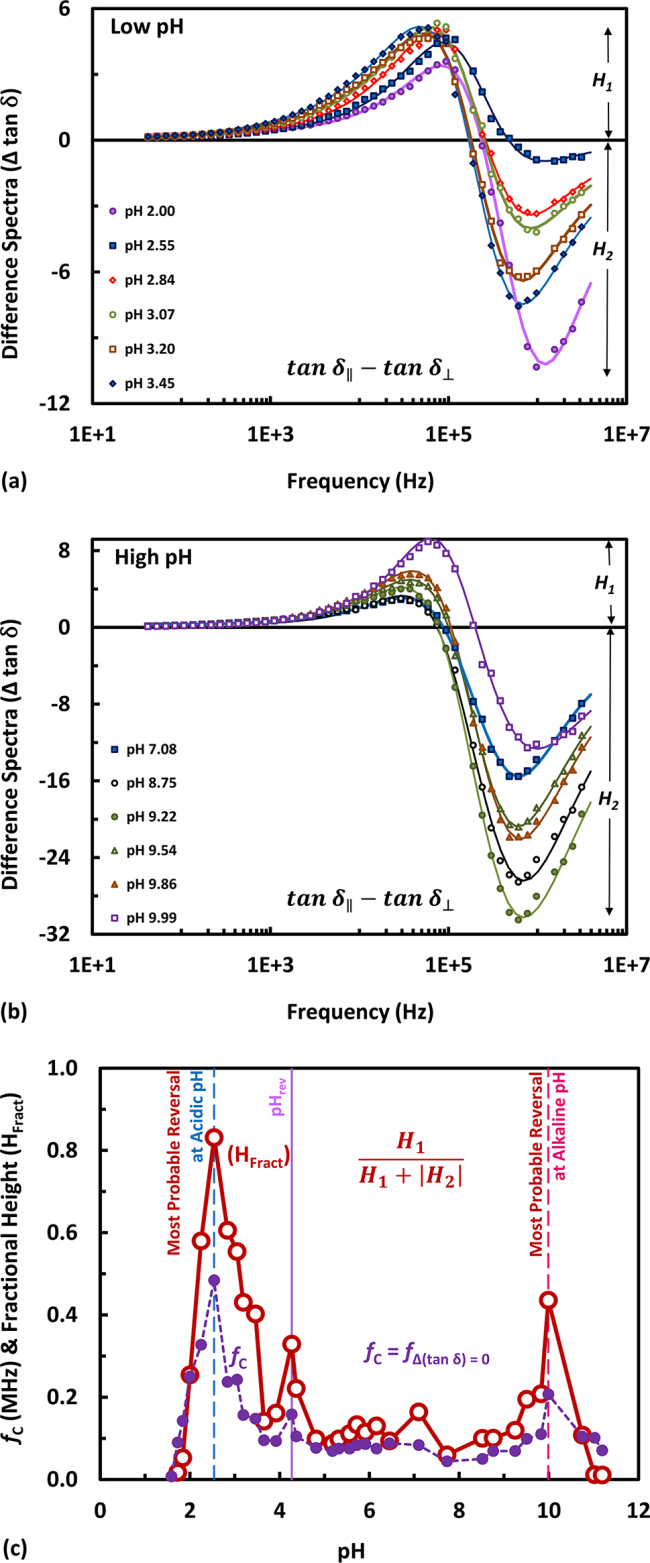
Difference spectra (Δtan δ) of PM. The data of (tan δ)_⊥_ is subtracted from that of (tan δ)_||_ at various pH values and some examples are shown in (**a**) at low and in (**b**) at high pH’s. The lines in (**a**) and (**b**) are due to Lorentzian fitting into two peaks. In addition, three pH sets are depicted in Fig. S6, Fig. S7 and Fig. S8 in the “Supplementary Information”. Some features of the biphasic (Δtan δ) spectra in dependence of pH are shown in (**c**). These features are the crossover frequency (*f*_C_) and the fractional height (H_Fract_ = H_1_/[H_1_ +|H_2_|]) of (Δtan δ) spectra; both are derived from the fitting carried out in (**a**) and (**b**). Both lines in (**c**) display the two peaks nearly around the same pH’s, as stated previously in Fig. 1c, marked by vertical dashed lines, in addition to the pH_rev_ marked by vertical solid line.

The dielectric absorption spectra (tan δ) determined at orthogonal directions (tan δ_||_ and tan δ_⊥_) have been depicted in Fig. 3a and Fig. 3b, in the form of difference spectra, Δtan δ, where Δtan δ = tan δ_||_—tan δ_⊥_, at low and high pH, respectively. Other pH sets of the difference spectra were depicted in Fig. S6 up to Fig. S8 in the “Supplementary Information”. The biphasic spectral differences were fitted to the Lorentzian function with two peaks (one positive of height H_1_ and one negative of height H_2_). This could be performed according to Eq. 5 comprising two Lorentzian components superimposed on a constant function. Two spectral features, viz*.* the fractional height (H_Fract_) of the biphasic spectra and the crossover frequency (*f*_C_), have been represented in Fig. 3c. It is not surprisingly here to find a second crossover frequency (*f*_C_), as seen in Fig. 3c, exhibiting pH dependences in a similar fashion to those shown in Fig. 1c, regarding the same three peaks. It based on rational reasoning that the spectra of tan δ are proportional with the permittivity. Therefore, the crossing of both comes in consistence. What is rather surprisingly here is that (H_Fract_) has ruled out the same conclusion respective to the three reversal peaks almost at the same three reversal pH’s (i.e. at discrete pH’s), as evident from Fig. 1c and Fig. 3c. It does mean that there is a consistency between the pH dependence of both (H_Fract_) (i.e. representing the ordinate of the biphasic spectra) and the crossover frequency (*f*_C_) (i.e. representing the abscissa of the biphasic spectra) at these discrete pH’s. This may underscore the significance of the three reversal pH’s in PM in terms of its crystalline structure on the one hand, and in terms of the anisotropic property of PM on the other hand. Changes occurring in the latter could reflect structural changes as follows. Surely, in fundamental research, the knowledge of structural variations can be of primary interest. Here, the study focuses, therefore, on the anisotropic property of PM, by virtue of being correlated with structural aspects of PM, i.e. molecular components of PM (bR-monomers, bR-trimers, and lipids) form asymmetric assemblies, which underpin its dielectric anisotropic property, as the spatial assembly of PM relies on the spatial distribution of the permittivity tensor. However, the biphasic property of circular dichroism^79–82^ could indicate about the assembling state of bR monomers, as well. For instances, in the early years after bR discovery, Muccio and Cassim^83^ did carry out extensive studies on the UV and visible circular dichroism of PM in a wide pH range (pH 2.4 – pH 12.6). Many researchers further employed circular dichroism of PM with dependence of pH^18,77,84,85^. Moreover, data were collected, in this respect, to investigate the structure of bR with dependence on pH, but with other techniques like IR^86,87^, NMR^88^, and diffraction at 2.3 – 2.7 A˚ resolutions^36^. These pH studies relying on techniques like circular dichroism, IR, NMR, and diffraction could be of importance to discuss some of the results presented here.

### Fluorescence spectra

**Fig. 4:**
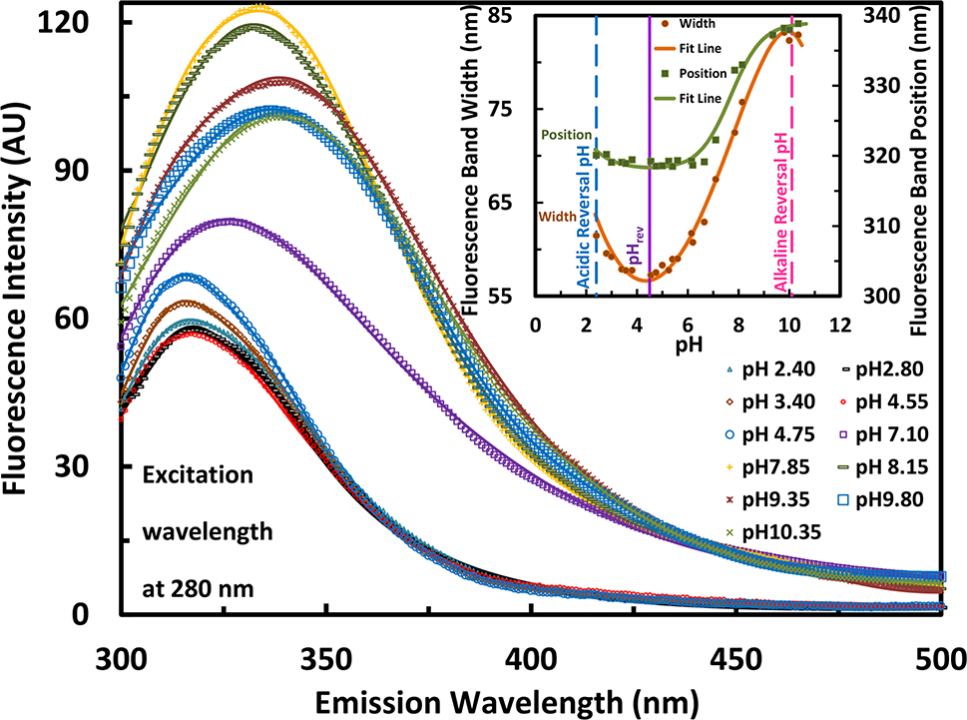
Fluorescence spectra of the isotropic state of PM in suspension. Typical examples of the pH dependence of fluorescence intensity in the range (300 – 500 nm) measured at an excitation wavelength of 280 nm are shown, while some features of the fluorescence spectra of the isotropic state of PM in suspension are shown in the inset. In addition, two pH sets are depicted in Fig. S9 and Fig. S10 in the “Supplementary Information”. The lines going through the data points are due to Lorentzian fitting of single band superimposed on quadratic background, from which the band width (in nm) and position (in nm) were derived. The pH dependence of fluorescence band width and position displayed a wide minimum centered around pH 4.5. The lines going through the data points in the inset are due to Henderson-Hasselbalch fitting to two global p*K*_a_’s around 2 ± 0.21 and 7.6 ± 0.16 (as an averaged values).

Because of the pH-induced changes in bR, the tryptophan and tyrosine environments can become less hydrophobic resulting in spectral feature changes that can reflect the effect of pH. In this respect, it should be pointed out that the tryptophan and tyrosine residues contribute to the interactions in the environment of retinal; the former to interactions with retinal, while the latter to interactions with the Schiff base linking the retinal to protein. The Tryptophan fluorescence is utilized as an internal indicator owing to being located near the retinal in bR. The fluorescence in bR owing to aromatic residues is subjected to quenching^89^ because of their close proximity to each other, on the one hand, and to the retinal, on the other hand, via the process of resonance energy transfer. Namely, the energy transfer should have pH dependence. This could be supported by the pH dependence of fluorescence measured in bR immobilized on carbon-nanotube sidewall^54^, from which the lack of close approach-to-retinal could be realized from the fluorescence from aromatic residues being almost transferred, instead, to the nanotube^54^, indicating a state of no quenching (i.e. not transferred to the nanotube in case of quenching). Moreover, there appeared in such studies^54^ that the dispersion of nanotube-bR biohybrid was stable in the pH range of (pH 4.5 – pH 9), which may be in the favor of measuring here the pH dependence of fluorescence. Therefore, beside to the passive electric measurements, fluorescence spectra had to be conducted, as well. The three reversal pH’s may be well-correlated to the features of fluorescence spectra measured for PM (but in suspension in the isotropic state). Typical examples of the UV fluorescence spectra are depicted in Fig. 4 at different pH values. Other pH sets are depicted in Fig. S9 and Fig. S10 in the “Supplementary Information”. The fluorescence spectra were well fitted to Lorentzian distribution composed of single band superimposed on a quadratic background according to Eq. 6. The value of the half-width at half-maximum (γ) were multiplied by 2 to get (*W*); the full width at half-maximum (FWHM) or simply the width. The spectral features (e.g. the width and position) of the UV emission, thus derived from Fig. 4, have been depicted in the inset. Both have displayed somewhat wide minimum centered at pH_rev_, while one peak has been displayed only at the alkaline side around pH 10.5 associated with almost no peak at the acidic side; the latter would appear but below pH 2.5. The emission-relevant pH dependences have been fitted to two global titrations (one acidic and the other alkaline) according to fitting of multiplicative combination (i.e. Equation 2) of Eq. 1 of Henderson-Hasselbalch.

### Logic gate Reasoning

**Fig. 5:**
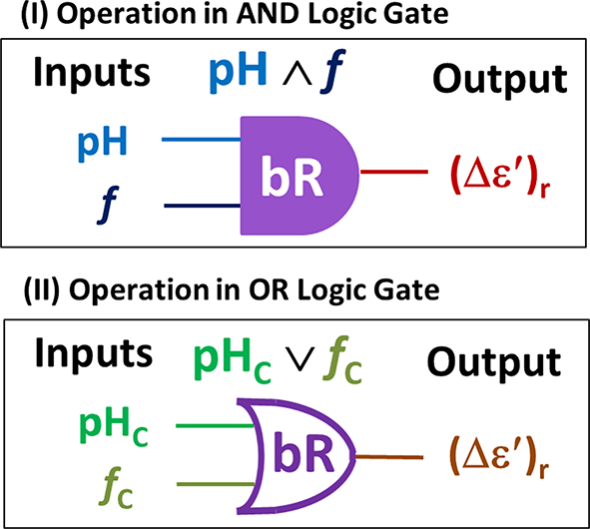
Block diagram of the Boolean logic gate realization in bacteriorhodopsin. The crossover pH_C_ (e.g. alkaline reversal pH) is the pH at which the anisotropy crosses from negative to positive. The crossover frequency (*f*_C_) (e.g. *f*_C High_) is the operating frequency at which the anisotropy crosses from negative to positive, as well. The AND operation of two inputs; (pH) and the frequency (*f*), which is written as (pH ∧ *f*) or as (pH . *f*) is given in (I). The OR operation of two inputs; pH_C_ and (*f*_C_), which is written as (pH_C_ ∨ *f*_C_) or as (pH_C_ + *f*_C_) is given in (II). While the interpretation of both operations in the logic theory is given here symbolically in (I) and (II), the truth table of both operations is given in Table 1.

An interest has been generated, from all the above observations, to focus on an impact of both pH and frequency dependences of dielectric anisotropy. A significant logic gate-based realization could be rewarded from the reversal of the dielectric anisotropy above both the crossover frequency (*f*_C_) and the crossover pH (pH_C_) in bR, which may be a candidate for future applications in bioelectronics and sensing technology according to the introduced concept focusing on anisotropy given in the “Introduction Section”. There are medical applications that are directly of pH relevance (or so indirectly through the level of protons relevant to the dissolution of CO_2_ gas). For instances, detection of biomarker proteins and pathogens plays central role in medical diagnostics. However, pH sensing has proved its applicability in technology; for review see Kieninger *et al*^90^. From conceptual standpoint being realized from the present measurements, the (pH) and the operating frequency (*f*) can be constructed in an AND logic gate, while the crossover pH (pH_C_) and the crossover frequency (*f*_C_) can be constructed in an OR logic gate, as can be indicated from the logic realization^91^ given in the block diagrams, shown in Fig. 5 and Table 1.

**Table 1:** Truth table of “AND” and “OR” logic gate operations in bacteriorhodopsin.

AND^a^ (Boolean Multiplication)						OR^b^ (Boolean Addition)					
Inputs				Output		Inputs				Output	
pH		*f*		(Δε^′^)_r_		pH_C_		*f*_C_		(Δε^′^)_r_	
0	Below pH_C_	0	Below *f*_C_	0	Not Reversed	0	NotAt pH_C_	0	Not At *f*_C_	0	Not Zero^c^
0	Below pH_C_	1	Above *f*_C_	0	Not Reversed	0	Not At pH_C_	1	At *f*_C_	1	Zero^c^
1	Above pH_C_	0	Below *f*_C_	0	Not Reversed	1	At pH_C_	0	Not At *f*_C_	1	Zero^c^
1	Above pH_C_	1	Above *f*_C_	1	Reversed	1	At pH_C_	1	At *f*_C_	1	Zero^c^

^a^In AND logic gate, the logic digit “0” stands for “Below pH_C_” meaning that the operation is at pH < pH_C_, whereas “1” for “Above pH_C_” meaning that it is at pH > pH_C_; the same holds for *f*_C_, as well. Note, for the output, that the logic digit “0” means no reversal of anisotropy occurred, while “1” means that there occurred a sign reversal.

^b^In OR logic gate, the logic digit “0” stands for “Not at pH_C_” meaning that the operation is at pH ≠ pH_C_, whereas “1” stands for “At pH_C_” meaning that it is at pH = pH_C_; the same holds for *f*_C_, as well. Arithmetically, at pH = pH_C_ and**/**or *f* = *f*_C_, the anisotropy factor (Δε′)_r_ equals zero; otherwise it does not equal zero. Logically, the digit “0” means here that the anisotropy is “Not Zero”, while “1” means that the anisotropy is “Zero”.

^c^Be careful not to be confused with the arithmetic zero and logic zero.

## Discussion

### The reversal correlates to acidic and alkaline disassembly of bR crystal

Changing of the crossover frequency, at which the anisotropy reversal occurs, with changing pH may be brought about by underlying consequences of the pH-induced structural changes in bR. Several literature observations could support correlating such reversal in the acidic and alkaline forms of bR to the disordering or disassembly of the crystalline structure of bR as follows:

1. The positive visible band in the circular dichroism reverses its sign at pH 2.4 indicating changes in the environment of the retinal due to some ionizable group titration^83^. This might be in the favor of the present acidic reversal of dielectric anisotropy around pH 2.5. As to the alkaline reversal of dielectric anisotropy around pH 10.5, drastic changes in the polarization anisotropy were reported to occur^72^, in the pH range of (pH 9 – pH 11) relative to the neutral suspension, indicating changes in the structure of PM itself, in addition to an increased rotational mobility of the protein, implying disassembly of PM within this alkaline pH range^72,83^. These changes resulted in disordering of bR crystal, which was found to be of less pronounced assembly at pH ≥ 10^71^. It is reasonable to suggest that this disassembly of PM^36,92^ might be correlated to the observed reversal of dielectric anisotropy at the alkaline reversal pH around 10.5.
2. Based on the previous point, it is not surprisingly therefore to find that the temperature of the main melting of PM had to be decreased from 96 **–**100 °C at neutral pH to 60 °C at both acidic pH (between pH 1.4 and pH 2.8)^77^ (Fig. 1 therein) and alkaline pH (above pH 10)^78^ (Fig. 2 therein). Overall, the lowering in the transition temperature to 60 °C (i.e. the acid blue form) could be understandable in terms of (α_II_ **–**α_I_) helical transition for both acid blue^87^ (around pH 2.5) and alkaline reddish^93^ (above pH 10) forms of bR.
3. Being this lowered transition temperature associated with lack of the reversible premelting transition belonging to the crystal lattice (e.g. in the acid blue bR) can be evident to some stabilization being offered to PM by the crystal lattice itself, as the lattice does melt usually within the range of 74 – 80 °C, i.e. lack of such lattice transition (or premelting) may rationalize such disordering of the PM hexagonal crystalline structure, which might be correlated to the reversal (or loss) of dielectric anisotropy observed in both forms.
4. This disordering in crystallinity might favor both the loss of ellipticity at alkaline pH > 11 and the prominent changes observed in the far UV region (together with sign reversal of the positive visible band) of the circular dichroism of bR at acidic pH of 2.4^83^, implying changes in PM assembly.
5. Changes in the tertiary structure of protein, in low range (pH 2.4 **–** pH 5) and high range (pH 8.5 – pH 11.8) of pH, may underlie this PM disassembly^83^.
6. The disordering might be understood in the view of the changes in the bR crystal size, whereby a shrinkage and an expansion in the bR crystal lattice, at low pH (pH 2 **–** pH 3) and high pH (pH 9 – pH 10), respectively were observed in diffraction studies^36^. However, the crystal becomes deteriorated (above pH 12)^73^.
7. The so-called proton release group exhibited changes^36^ at the acidic and alkaline reversal pH’s, as well. The proton release group in bR is a hydrogen-bonded structure comprising of Arg82 and two Glu’s at 194 and 204, together with water. It exists in the pathway of proton toward the extracellular surface^29,36^. Owing to the strong interaction of the hydrogen bonded glutamates (194 and 204), the proton release group seems to be a key determinant in the stabilization^36^ of bR crystalline lattice in the pH range of (pH 3–pH 10). Outside this range, the structure of the proton release group was broken both at the acidic side below pH 3 (where both key glutamates are protonated) and at the alkaline side above pH 10 (where both key glutamates are deprotonated)^36^. The broken proton release group, together with changes in the crystal size stated above in the previous point, below pH 3 and above pH 10, might bring about such reversal of anisotropy observed here around pH 2.5 and pH 10.5, respectively, as shown in Fig. 2 (inset) in addition to Fig. S5 (inset) in the “Supplementary Information”. The proton release group seems, in general, to be an essential determinant to both acidic and alkaline reversal of the dielectric anisotropy. Additionally, it seems likely that some correlation of the crystalline structure of bR with such hydrogen-bonded network of the release group exists in terms of the reversal pH’s.
8. These influences exerted by pH-induced structural changes in bR may be viewed from the consequent changes occurring in the protein emission. The influence of pH on bR can be monitored from the fluorescence spectral characteristics that reflect conformational and configurational changes occurring in both protein part (i.e. UV-fluorescence)^54^ and non-protein part (visible fluorescence)^89^ of bR. The protein fluorescence is originated due to UV-excitation concerning the aromatic residues (tyrosine and tryptophan). Because of the close proximity to retinal resulting in fluorescence quenching, the spectral shift, together with the spectral width, can be rather more conclusive in reporting pH effects to indicate that structural changes of the protein, in addition to its interactional changes with the retinal, might be occurred considerably at the acidic and alkaline reversal pH. For an instance, a peak at the alkaline reversal pH around 10.5 in the pH dependence of the fluorescence spectral features (band width and position) does imply specific aromatic environment of tyrosine and tryptophan in the alkaline form of bR^94^.
9. Both acid (blue)^95^ and alkaline (purple)^93^ forms of bR contain two retinal conformers (all-*trans* and 13-*cis*). What is more, both exhibited nearly similar increase of (5°)^83^ and (5.5°)^96^ in the direction of the transition dipole moment of the retinal out of the membrane surface for the alkaline (purple) and deionized (blue) forms of bR, respectively, that necessitates geometrical imposes on the protein contributing to such crystal disassembly^18,36,97^.

All the above observations do point to a correlation seemingly found between the reversal of dielectric anisotropy at both forms of bR (acid blue and alkaline purple) and the crystal disordering (or even crystallinity loss), at the reversal acidic and alkaline pH on both sides of the pH_rev_, respectively, in the view of the essence of crystalline lattice. This picture (refer to points 1 **–** 9) may contend to describe both acidic and alkaline state of bR as a molten globule-like state^98^, which could host well logic gate operation in bR^99^. This would be justified only if the reduced melting temperature^77,78^ (refer to point 2) in bR would be proposed to be ensued from gradual unpacking of the side chains of amino acids (i.e. tertiary structure of the protein) (refer to point 5), initiated in both acidic and alkaline states of bR^83^, resulting in disordering in the crystalline structure of PM, which could be supported by, again, the ellipticity loss (refer to point 4)^83^, concomitant with (α_II_ **–** α_I_) helical transitions (refer to point 2) observed in both acidic^87^ and alkaline state^93^ of bR.

Correlating the reversal to crystalline disassembly most probably mediated by the lipid-protein contact^71^. It is well known that the phospholipids are located on the cytoplasmic side, whereas the glycolipids on the extracellular side of PM^71^. Among the phospholipids, there appeared that both phosphatidylglycerophosphate methylated (PGP-Me) and phosphatidylglycerosulphate (PGS) are crucial for maintaining the hexagonal crystalline lattice of bR^100^. What is more, the hexagonal lattice was exhibited well only when either of these phospholipids was existed in bR reconstituted-lipid system^100^. For instance, the major phospholipid (PGP-Me) proved crucial in maintaining the assembly of the crystalline lattice, as it connects two adjacent bR monomers. By virtue of its outstanding diffraction pattern^12^, the major triglycosyl lipid (S-TGA-1), located in the intra-trimeric and inter-trimeric spaces (one per trimer space)^101^, proved crucial, as well^12^.

### The reversal occurs outside the pH range of proton pumping

Worthwhile to note, firstly, that the polarity of both forward (upon switching light on) and reverse (upon switching light off) currents generated in bR was found to reverse directions at the bR isoelectric point (at about pH 5.8)^24–26^, which is presumably equivalent to the pH_rev_ (at about pH 5)^21–23^. Again, one would be not confused with the reversal of surface charge asymmetry of PM occurring at pH_rev_ and such reversal of the anisotropy occurring outside the pH range of proton pumping, to which there appear several literature observations favoring its pertinence to occur outside such pH range as follows:

1. The relationship of the forward to reverse current ratio versus pH^24^ was found to be so linear solely within the pH range of (pH 3.5 – pH 9) that could enable utilizing bR as pH sensor^24^.
2. Similar pH range (pH 4 – pH 10) did contend, in a more recent study^60^, to the stationary level of bR output (i.e. photocurrent or proton pumping) within such pH range to the extent that allowed bR to be used as a pH biometer, as well^60^.
3. Stability of the dispersion of bR-carbon nanotube bio-hybrids within the pH range (pH 4.5 – pH 9) in fluorescence studies^54^ might imply the bio-nano interactions (i.e. between bio- and nano-materials) of the bR immobilized on the sidewall of the carbon nanotube being influenced by the reversal of dielectric anisotropy in bR occurring outside such pH range, i.e. it is a clue to the essential aspect of anisotropy of biomaterials in bioelectronics. However, the membrane capacitance is useful tool to indicate successful incorporation of bR into planner lipid bilayer^102^. It does mean that changes in permittivity favoring the anisotropy to reverse may imply the instability of the bio-nano interactions to appear.
4. Reducing proton release outside such pH range of (pH 3 – pH 10) comes consistent not only to the proton release group in bR to appear broken outside such range^36^, but also to several observations did occur outside such pH range (refer to points 1 **–** 9 in the previous section). The consequence of these observations did appear in the proton pumping activity of bR, as the low pH^18,61^ and high pH^61^ forms of bR exhibited considerable reduction in the proton pumping. Electric studies showed that the bR photocycle, proton release and proton uptake are correlated with electric events in bR^61,103–106^. In addition, relevant anisotropic electric studies on PM^75^, indicated that there occurred dynamic changes in its permanent electric dipole moment (i.e. properties across PM being electrically anisotropic) during the bR photochemical cycle. Therefore, it is reasonable to anticipate that the proton pumping, which results in a photocurrent signal, should be in keeping with the permittivity function in bR.

Actually, the present pH dependence of the orthogonal dielectric permittivity, as demonstrated in Fig. 2 (in addition to Fig. S5 in the “Supplementary Information”), somewhat resembles the pH dependence of area of the photocurrent signal^61^ (strictly of the B2 component) in bR (see the representation number 7 therein^61^), but in the reciprocal form. This B2 component belongs to the proton release during L-M transition during the photocycle of bR. A considerable drop in the area of B2, at both low and high pH, is a manifestation to the drop in the proton release, which was found to occur with p*K*_a_ of 2.8 and p*K*_a_ of 9.1, respectively^61^. However, two groups were found to titrate at p*K*_a_ of 3.5 and p*K*_a_ of 10 in bR^37,38^. These p*K*_a_’s are in a close agreement to the respective p*K*_a_’s presented here, concluding unambiguously that the reversal in anisotropy might pertain to occur at those pH’s that are outside the pH range of the proton pumping. Another point of interest is that a drop in the proton pump with p*K*_a_ identical to the p*K*_a_ of the transition of slow M intermediate (M^s^) to the fast one (M^f^), at high pH, may be in the respect of existing two parallel photocycles of bR but with different aspects regarding the reversal dielectric anisotropy. In other words, the transition from M^s^ to M^f^ may be relevant to the transition from negative to positive anisotropy at the alkaline reversal pH around 10.5, where the M^f^ becomes significant at high pH above 10^107^. If so, M^s^ would be of negative anisotropy, while M^f^ is of positive anisotropy. This might signify two parallel photocycles of different anisotropic sign.

### The reversal is based on logic gate operation

The protein bR is best suited to represent the biology-electronics integration^2–4,108^. Nowadays, using the Boolean logic gates has smart progresses in several biological topics^109^. The results here may be exploited in technical applications based on Boolean reasoning as indicated schematically in Fig. 5 and Table 1. The working principle of such realization of employing bR as a logic gate relies on a change occurring in the sign of dielectric anisotropy, displayed by bR when it interacts with AC electric field above both (i.e. AND) its crossover frequency (*f*_C_) and its crossover pH (pH_C_). Such a reversal in sign, from a practical point of view, can be detected as a change, for instance, in a voltage level quantized into only two reasoning levels. This reasoning underpins the Boolean AND logic realization^91^ of defining the crossover pH (pH_C_) as the pH (e.g. alkaline reversal pH) at which the anisotropy reverses its sign provided that the operating frequency be also above the crossover frequency (*f*_C_). As input values to the Boolean operator AND here, pH’s above (pH_C_) correspond to logic digit 1, while those below it to logic digit 0. The same logic realization can be given to operating frequencies above and below the crossover frequency (*f*_C_) (refer to Fig. 5 and Table 1). At the output, the logic digit 1 is represented by reversed anisotropy, while the logic digit 0 by not-reversed one, this is for an AND operation in bR as follows:

8 $${\left({\Delta \upvarepsilon {\prime}}\right)}_{{\text{r}}}= \left\{\begin{array}{lll}1\quad if\,and\,only\,if \quad pH=1 f=1 \\ 0&\quad if\quad otherwise \end{array}\right.$$

The same spoken can be given to an OR operation but with different inputs and different output as follows:

9 $${\left({\Delta \upvarepsilon {\prime}}\right)}_{{\text{r}}}= \left\{\begin{array}{lll}0&\quad if\,and\,only\,if&\quad{\text{pH}}_{{\text{C}}}=0 {f}_{C}=0 \\ 1&\quad if&\quad otherwise\end{array}\right.$$

For these different inputs, pH “At” the crossover pH (i.e. at pH_C_) corresponds to logic digit 1, while those “Not At” such (pH_C_) correspond to logic digit 0. The same is self-explanatory for an operating frequency “At” (or “Not At”) the crossover frequency (*f*_C_) (refer to Fig. 5 and Table 1). At the output, logic digit 1 is represented by “None-Zero”-anisotropy, while logic digit 0 by nullified one (i.e. “Zero”).

In summary, a sensing system of bR based on the AND and OR logic gate using two inputs, viz*.* (pH and *f*) and (pH_C_ and *f*_C_), respectively, may be realized to be integrated in other systems^109^, as well. In the present case, the AND logic gate results in an output sensing only in the presence of “True” value for both inputs (pH and *f*) but not when either or neither input is “True” (see Eq. 8). The OR logic gate results in an output sensing only when either one of the inputs (pH_C_ and *f*_C_) or both is “True” but not when both is “False” (see Eq. 9). While this realization was for employing bR as a non-optical logic gate (i.e. electrically tunable), the same bases can be realized for bR to be employed rather as an optical logic gate (i.e. optically controlled) in photonic technology^110^, in case the bR photocycle would be intended, as well. Namely, the accord of being both forms of M-intermediate of bR (Fast or Slow) of different signs in its dielectric anisotropy (Positive or Negative), as suggested above, may be exploited logically. Utilizing the dynamics of M-intermediate of bR^111^ in photonic logic gate may be considered also in the respect of such anisotropy reversal. However, the all-optical logic gate reasoning (i.e. controlling light by light) based on bR^109^ has witnessed remarkable progresses in technology-relevant researches relying on the well-established understanding of the bR photocycle. At the same time, understanding the bR photocycle in the aspect of being that the bR-intermediates may possess dielectric anisotropy of different signs may be worthwhile for further progresses in future researches in photonic technology, as well.

## Conclusion

The bR was explored as a dual frequency biomaterial, which can find its potential applications in technology based on the introduced reasoning of employing bR as a logic gate. This characteristic was imparted to bR by virtue of its crossover frequency, which exhibited most probable state of reversal in the dielectric anisotropy at two discrete pH’s (around pH 2.5 and around pH 10.5) respective to the acid (blue), and alkaline (purple) forms of bR, respectively. The reversal in dielectric anisotropy occurring outside the pH range of proton pumping seems to be correlated to the crystalline disordering in bR resulting in reduction of the proton pumping. These forms of bR (acid blue and alkaline purple) may be of technical relevance, as well. Lastly, a future perspective of engineering bR in the manner that may enable exploiting the anisotropy reversal for purposes of reducing the proton pumping (i.e. switching off the output photocurrent) at desired technological conditions may not be elusive.

## Electronic supplementary material

Below is the link to the electronic supplementary material.

Supplementary Material 1

## Data Availability

“All data generated or analysed during this study are included in this published article”.
