# Supplementary material for: Bacteriorhodopsin of purple membrane reverses anisotropy outside the pH range of proton pumping based on logic gate realization
Source: Sci Rep. 2024 Nov 27;14:29452. doi: 10.1038/s41598-024-80512-0 (PMC11603030; doi:10.1038/s41598-024-80512-0)
Supplement: Supplementary file 1 — Supplementary Material 1 [file 41598_2024_80512_MOESM1_ESM.pdf]

Supplementary Information

Fig. S1 (The 2nd pH set for anisotropy)

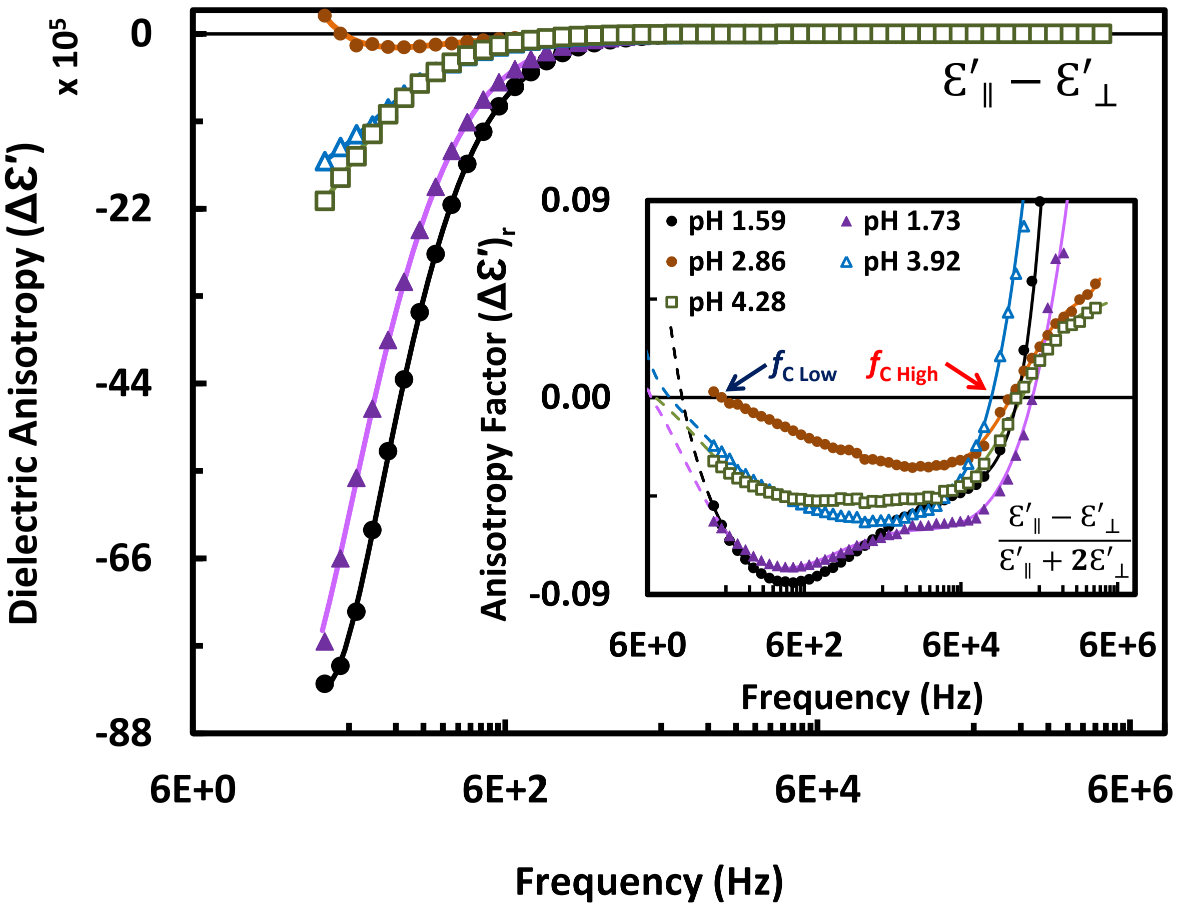

Fig. S1: The pH dependence of the crossover frequency ($f_{C\ Low}$) and ($f_{C\ High}$) at low and high frequency, respectively. The frequency dependence of the dielectric anisotropy (ΔƐ′) of immobilized PM for the 2nd pH set other than depicted in the manuscript. The inset represents the frequency dependence of anisotropy factor (ΔƐ′)$_r$, in accordance to the inset formula. The crossover frequencies ($f_{C\ Low}$) and ($f_{C\ High}$) are derived from the inset by polynomial extrapolation of the data back to cross the abscissa as indicated by the dashed back extensions. Note that the data at pH 2.86 is re-depicted here merely to be a reference for such data that crosses the abscissa with no extrapolation.

Fig. S2 (The 3rd pH set for anisotropy)

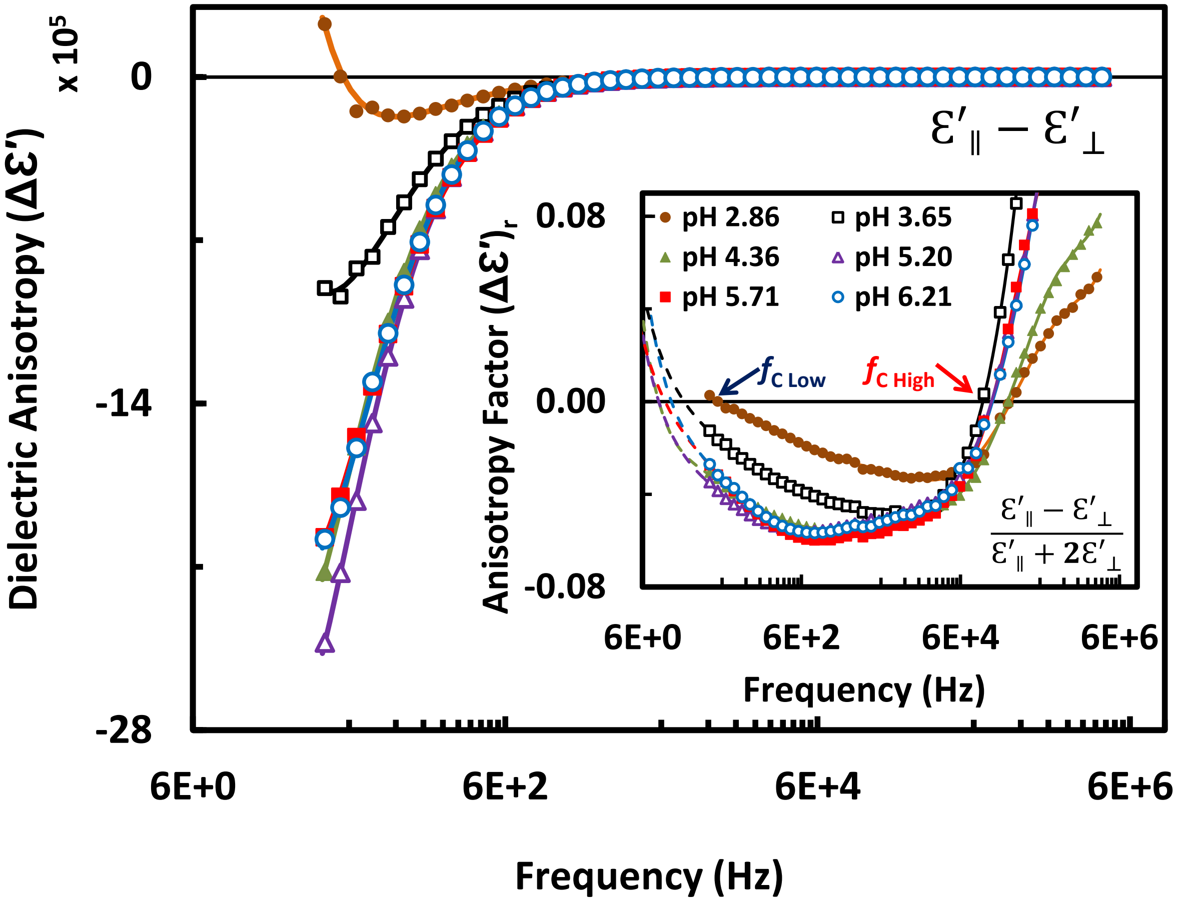

Fig. S2: The pH dependence of the crossover frequency ($f_{\mathrm{C\ Low}}$) and ($f_{\mathrm{C\ High}}$) at low and high frequency, respectively. The same caption of Fig. S1 could be given for the 3rd pH set.

Fig. S3 (The 4th pH set for anisotropy)

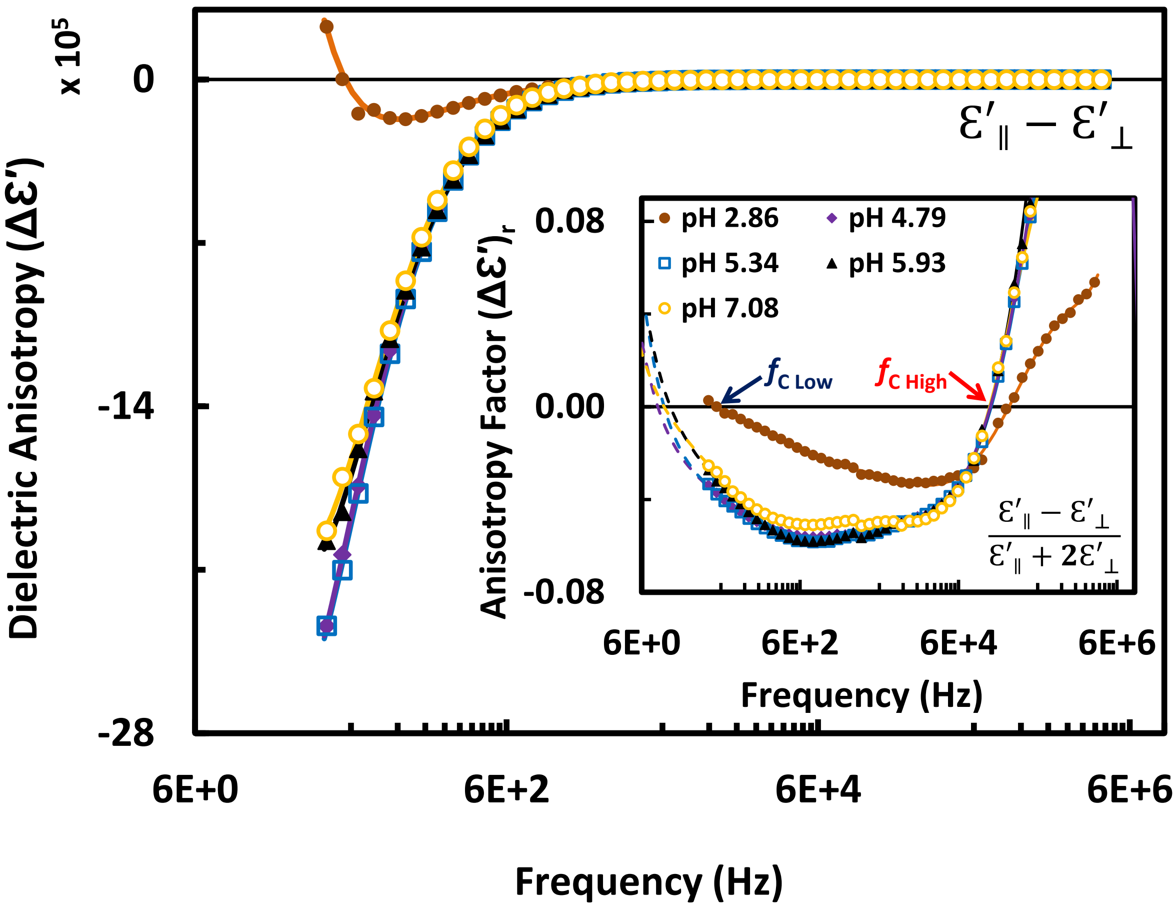

Fig. S3: The pH dependence of the crossover frequency ($f_{\mathrm{C\ Low}}$) and ($f_{\mathrm{C\ High}}$) at low and high frequency, respectively. The same caption of Fig. S1 could be given for the 4th pH set.

Fig. S4 (The 5th pH set for anisotropy)

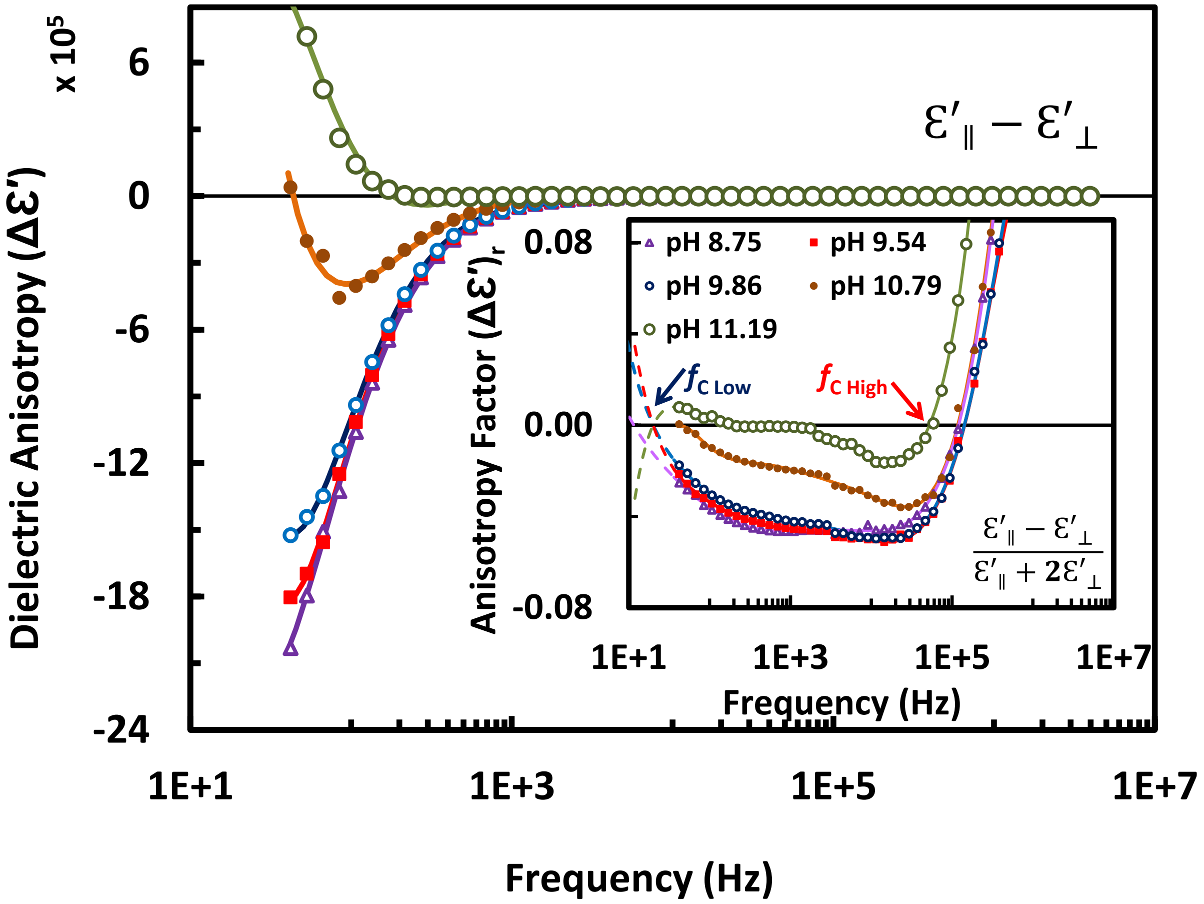

Fig. S4: The pH dependence of the crossover frequency ($f_{C\ Low}$) and ($f_{C\ High}$) at low and high frequency, respectively. The same caption of Fig. S1 could be given for the 5th pH set. Note the anomalous behavior of the trace at pH 11.19.

Fig. S5 (The orthogonal permittivity & anisotropy values are collected at intended single frequency at wide pH range)

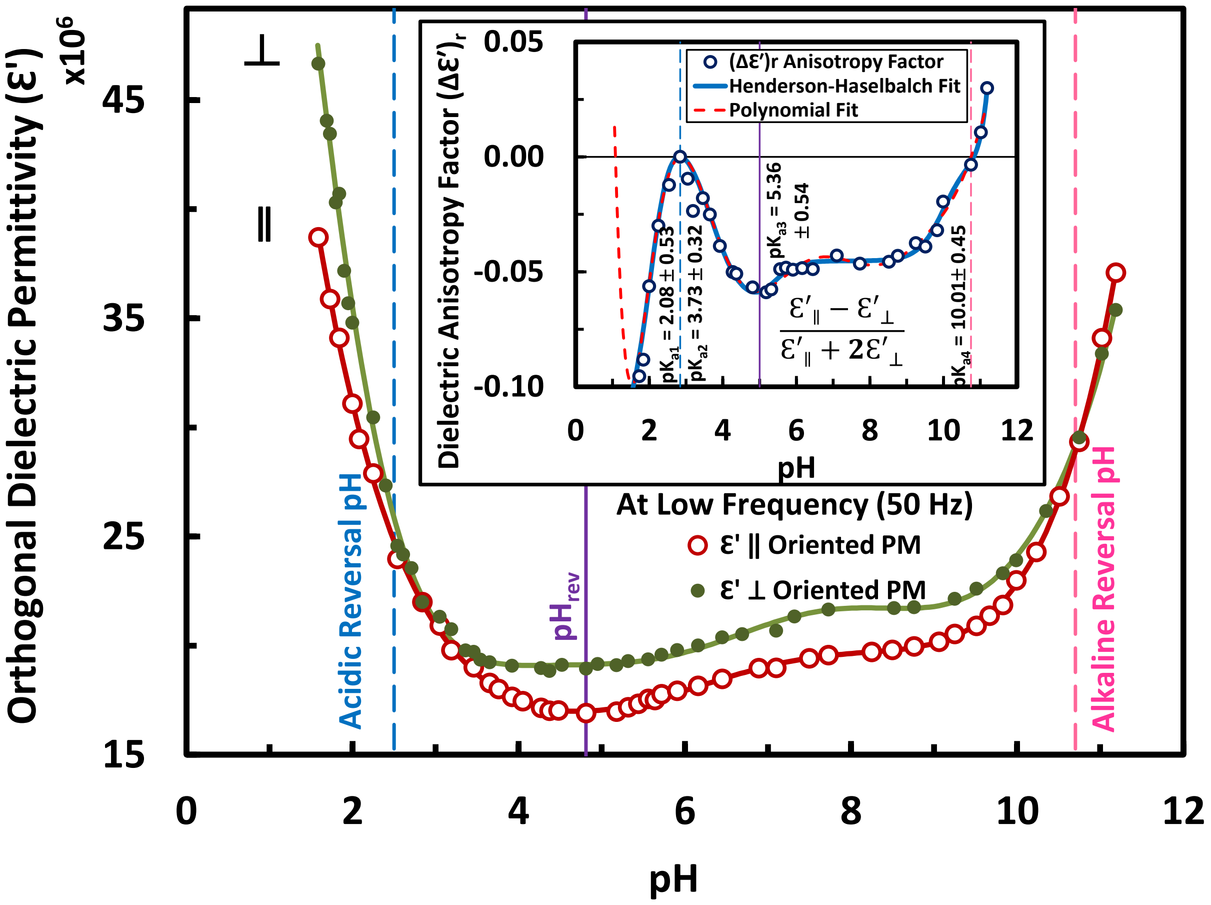

Fig. S5: The pH values at intended single frequency are depicted to represent the orthogonal permittivity and anisotropy collected from the previous figures (e.g. from Fig. S1 up to Fig. S4). The single frequency here is 50 Hz is intended, beside to the frequency 42 Hz in the manuscript, as the frequency relevant to the dispersion of permanent electric dipole moment of PM lies in the range 10 Hz - 100 Hz as well as both (50 Hz and 42 Hz) represent the maximum crossover frequency $(f_C)_{max}$ at the alkaline and acidic reversal pH's, respectively. The permanent electric dipole moment of PM is intended, as it mainly concerns the dielectric anisotropy.

Fig. S6 (The 2nd pH set for difference spectra of tan δ)

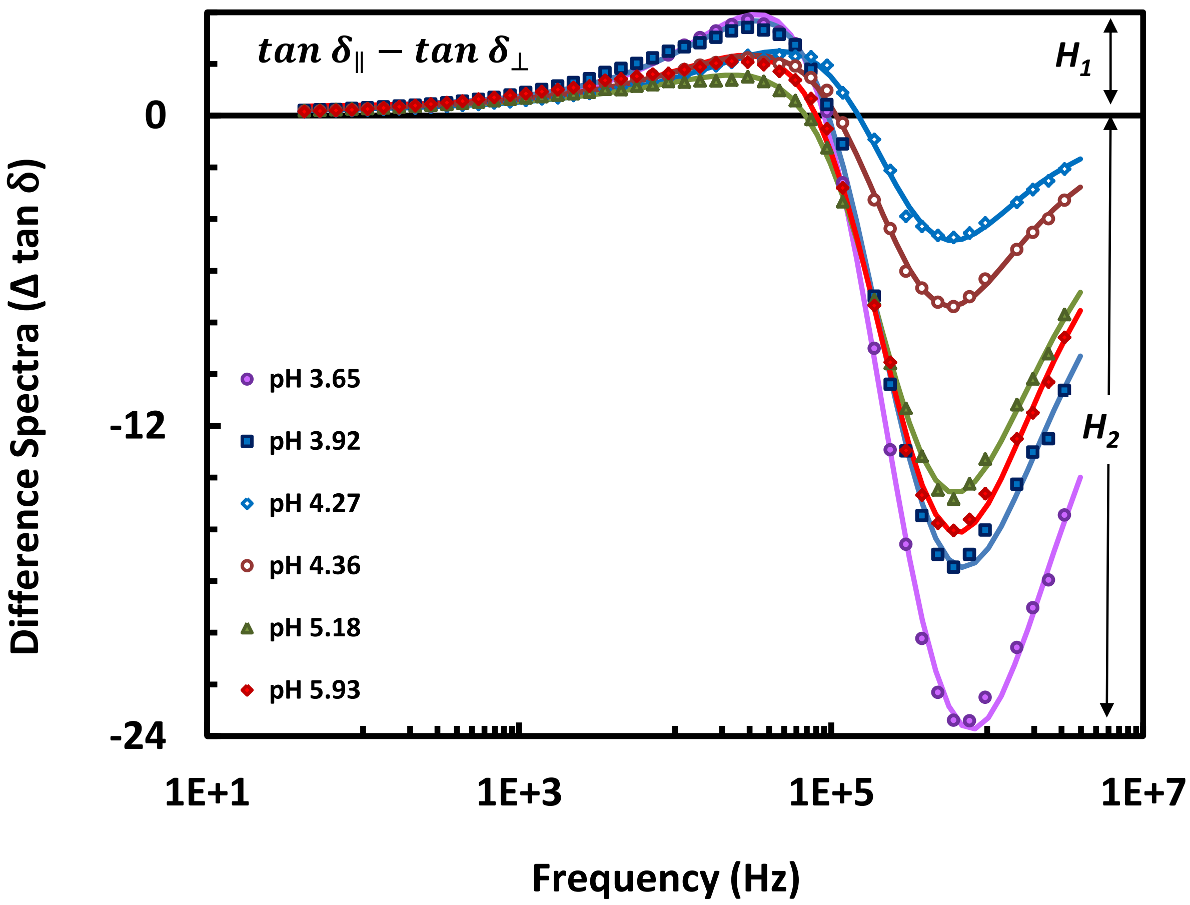

Fig. S6: Difference spectra of tan δ of PM for the 2nd pH set other than depicted in the manuscript. The lines are due to Lorentzian fitting into two peaks.

Fig. S7 (The 3rd pH set for difference spectra of tan δ)

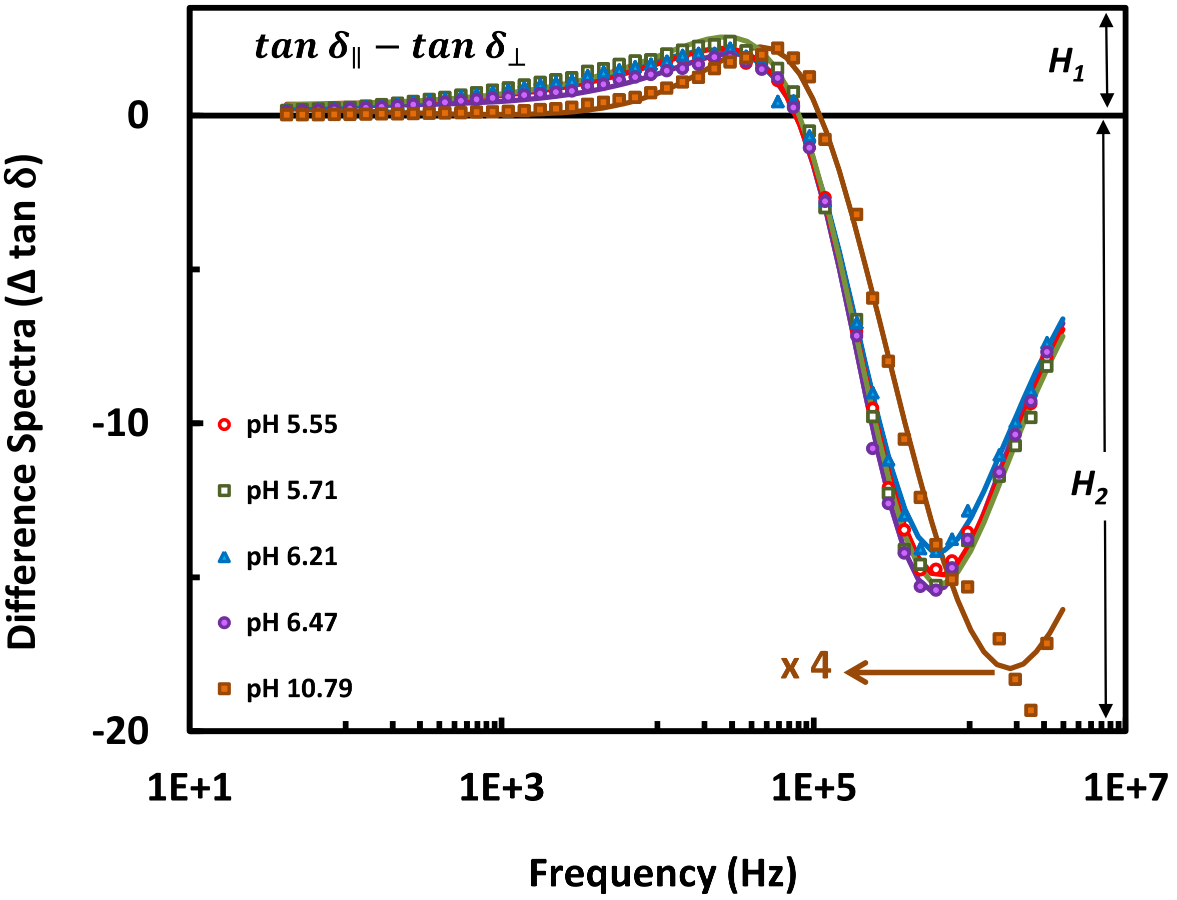

Fig. S7: Difference spectra of tan δ of PM for the 3rd pH set. The lines are due to Lorentzian fitting into two peaks.

Fig. S8 (The 4th pH set for difference spectra of tan δ)

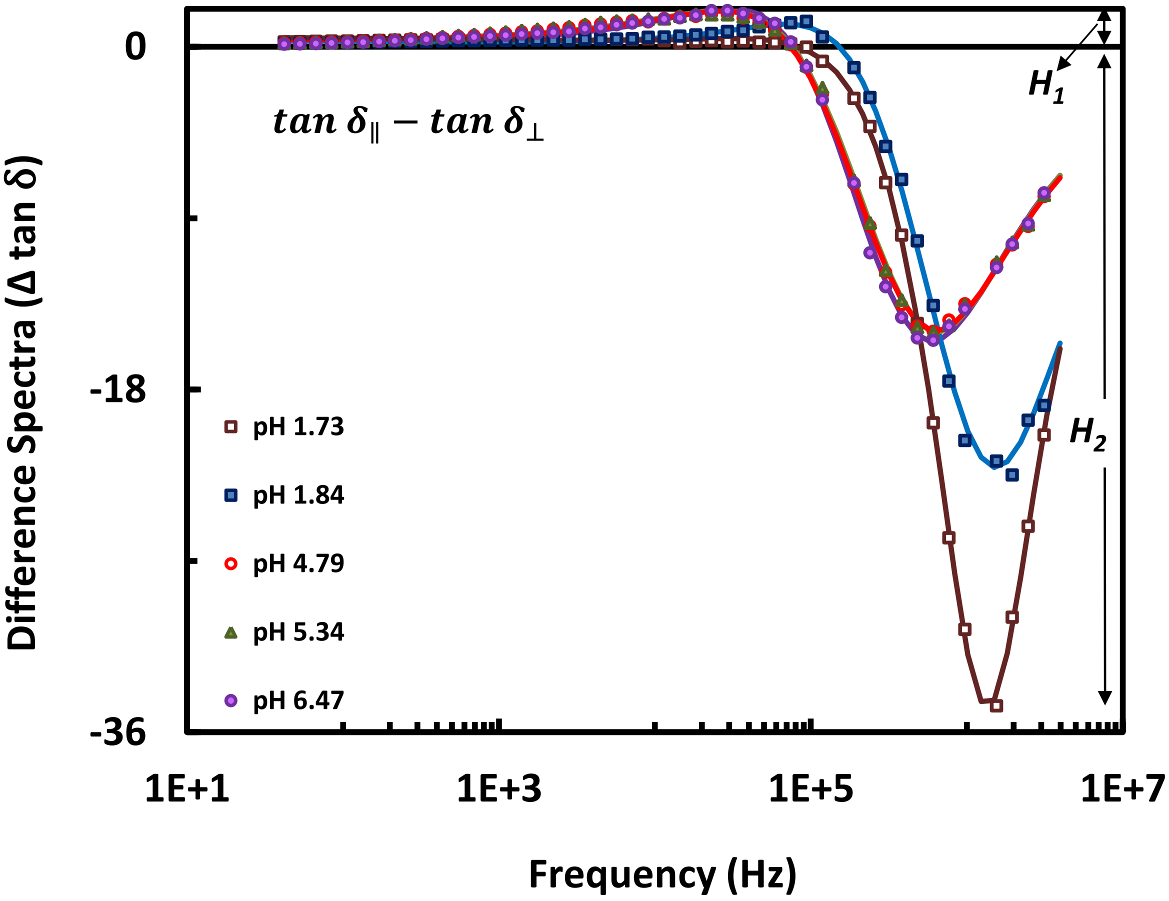

Fig. S8: Difference spectra of tan δ of PM for the 4th pH set. The lines are due to Lorentzian fitting into two peaks.

Fig. S9 (The 2nd pH set for fluorescence spectra)

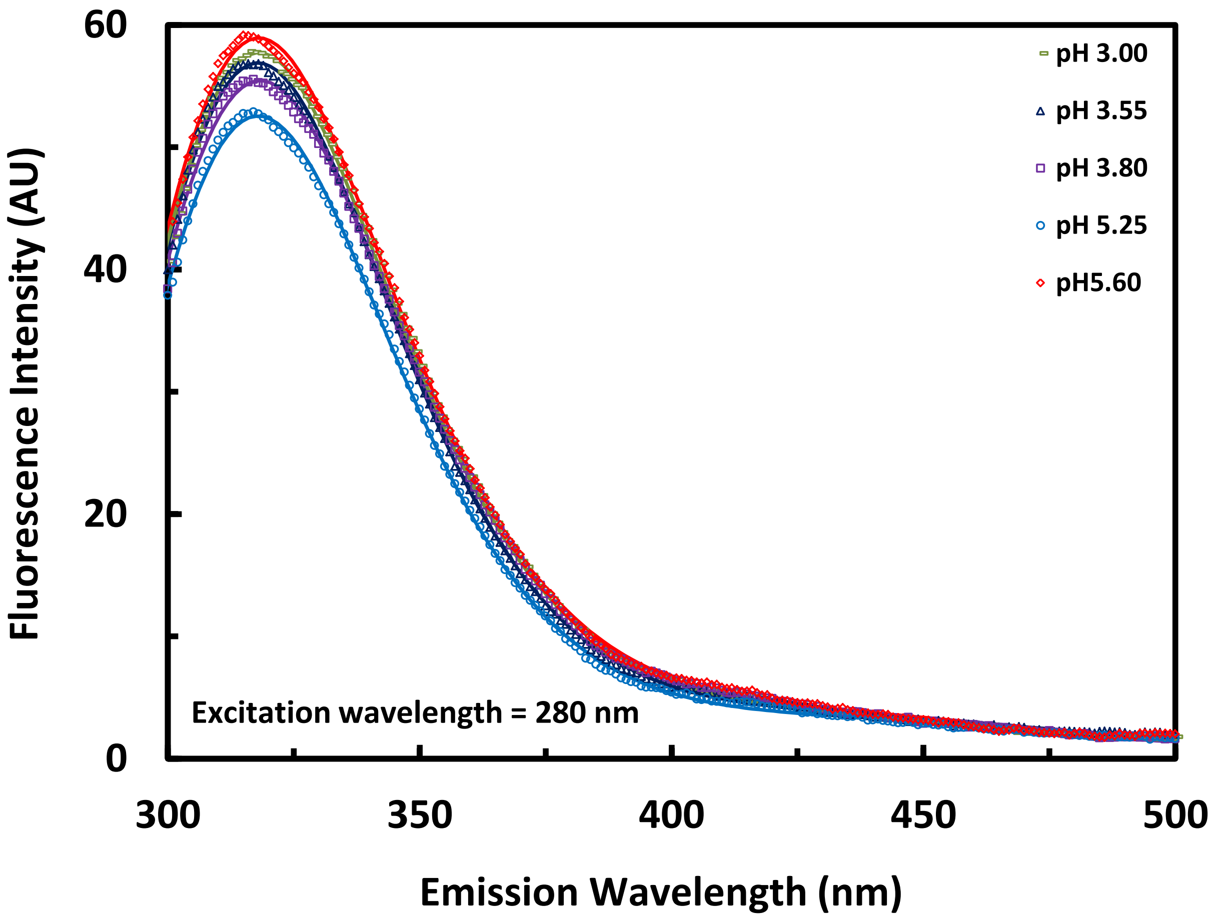

Fig. S9: Fluorescence spectra of the isotropic state of PM in suspension. The 2nd pH set other than depicted in the manuscript for the pH dependence of fluorescence intensity in the range (300 – 500 nm) measured at an excitation wavelength of 280 nm are shown. The lines going through the data points are due to Lorentzian fitting of single band superimposed on quadratic background.

Fig. S10 (The 3rd pH set for fluorescence spectra)

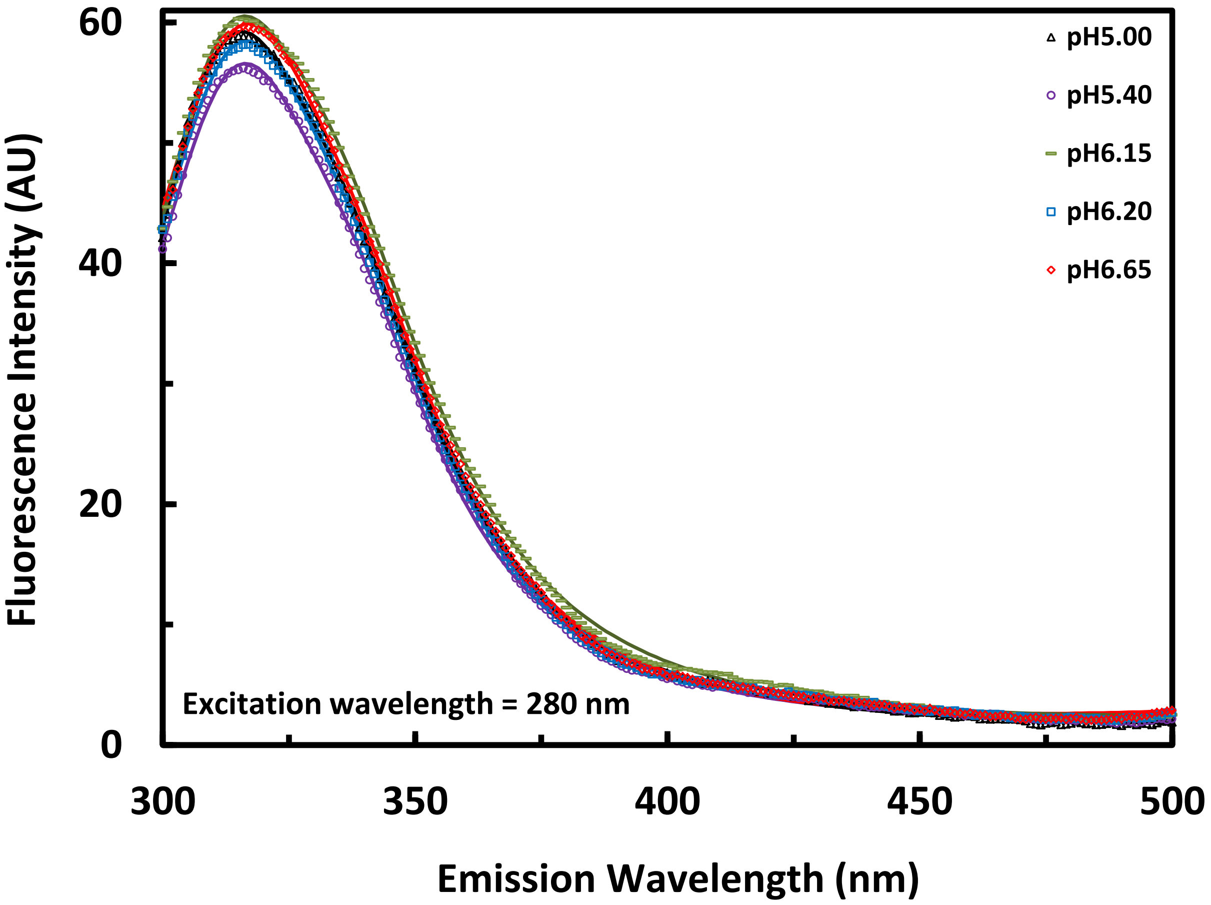

Fig. S10: Fluorescence spectra of the isotropic state of PM in suspension. The 3rd pH set of the pH dependence of fluorescence intensity in the range (300 – 500 nm) measured at an excitation wavelength of 280 nm are shown. The lines going through the data points are due to Lorentzian fitting of single band superimposed on quadratic background.
